# Valorisation of Insect Farming By-Products into Chitosan: Chemical Characterisation and Bioactivity for Veterinary Use

**DOI:** 10.3390/molecules31203782

**Published:** 2026-10-21

**Authors:** Carolina Ligeiro, Daniel Murta, Luísa Lima Gonçalves

**Affiliations:** 1 Egas Moniz Center for Interdisciplinary Research (CiiEM), Egas Moniz School of Health & Science, 2829-511 Caparica, Portugal; dmurta@egasmoniz.edu.pt (D.M.); lgoncalves@egasmoniz.edu.pt (L.L.G.); 2 Thunder Foods, 2005-332 Santarém, Portugal

**Keywords:** insect-derived chitosan, *Hermetia illucens*, *Tenebrio molitor*, biomaterials, veterinary medicine

## Abstract

Insect-farming by-products provide an alternative source for chitin and chitosan production, adding value to existing protein-production systems. This narrative review examines extraction, physicochemical characterisation and bioactivity from documented by-products of *Hermetia illucens* and *Tenebrio molitor.* Veterinary applications are discussed separately, drawing on studies using chitosan of other or unspecified origins to identify potential uses and the evidence needed for insect-derived materials. Exuviae, empty puparia and documented adult residues are distinguished from whole-insect materials used as methodological comparators. Chemical, enzyme-assisted and intensified processes produce materials with different degrees of deacetylation, molecular weights, residual components and recoveries; yields relative to biomass are distinguished from conversion yields relative to isolated chitin. Direct insect-derived evidence consisted mainly of in vitro antimicrobial, antioxidant, epithelial-response, haemocompatibility and degradation assays, with limited preclinical haemostatic testing. No veterinary clinical or field study using a defined insect-derived chitosan product was identified. Studies of wound management, tissue repair, delivery systems and dietary supplementation showed formulation-, dose- and species-dependent responses, without establishing equivalence between chitosan sources. Veterinary translation requires traceable sources, reproducible processing, validated characterisation, contaminant control and product-specific safety and efficacy studies. Environmental and economic benefits also require comparative assessment. Insect-derived chitosan therefore warrants further investigation as a veterinary biomaterial, with suitability demonstrated for each preparation and application.

## 1. Introduction

The rapidly increasing global market demand for chitin and chitosan across food, pharmaceutical, biomedical, and industrial applications has created an urgent need to identify alternative, sustainable sources [1]. Conventional production from crustacean-processing residues requires chemical inputs and management of extraction effluents; comparisons with insect-derived production must account for their residual origin [1]. Insect-derived chitosan production represents a viable pathway to meet this growing demand while providing farmers and insect breeders with additional revenue streams through value-added product recovery from their operations [2].

The black soldier fly (BSF, *Hermetia illucens*) is of particular interest because its farming systems generate cuticular residues (exuviae) alongside protein- and lipid-rich products. Primary studies have examined *H. illucens* exuvia, puparia and documented dead-adult residual streams. Puparia gave the highest chitin recovery among larvae, exuviae and adults in one within-study comparison, while composition differed substantially between streams [2].

Beyond BSF, research demonstrates that various insect sources including mealworms (*Tenebrio molitor*), crickets (*Acheta domesticus*), grasshoppers, and cicadas can serve as effective chitosan sources [3]. *T. molitor* studies have examined exuviae, documented breeding rejects and processing residues [4,5]. Extraction outcomes vary with material, developmental stage, pretreatment and analytical method. For example, reported *H. illucens* chitin yields include values from approximately 6–8% for one puparia protocol and 13.9–14% for another, while the literature also contains higher values obtained with different starting materials and calculation bases [6,7]. Available evidence attributes values above 35% to larval exoskeleton or comparable chitin-rich materials [2,8]. Chitin values are also method-dependent: ADF measurements can exceed ADF-ADL or acetyl-group measurements, whereas ADF-ADL and acetyl determinations may be more comparable [8].

This review focuses on the valorisation of documented farming and processing by-products of *H. illucens* and *T. molitor*. By-products are defined operationally as secondary material streams generated during insect rearing or processing, rather than insects harvested specifically for chitin or chitosan extraction. The materials considered comprise exuviae from both species, empty puparia from *H. illucens*, chitin-containing processing residues, and dead adults or rejected insects where their status as residual streams is documented.

Because terminology varies across studies, material names are standardised throughout this review according to their documented origin. “Exuviae” (singular: “exuvia”) denotes cuticles shed during larval moulting in either species. This category includes materials described as “sheddings”, “larval exuviae” or “larval exoskeletons” only where the source confirms that they are shed cuticles rather than exoskeletons isolated from harvested larvae. “Puparia” (singular: “puparium”) denotes the cases remaining after adult emergence in *H. illucens*, sometimes termed “pupal exuviae”, “pupal exoskeletons” or “cocoons”. Dead adults and rejected insects retain these designations and are reported separately.

Studies using whole insects harvested for extraction are retained as comparative evidence on extraction methods, developmental stages and material properties, but are distinguished from studies demonstrating by-product valorisation. Evidence from other chitosan sources is used to contextualise potential veterinary applications and is clearly separated from findings obtained with insect-derived materials. This review therefore links defined insect material streams and extraction conditions with polymer properties, bioactivity, the strength of veterinary evidence and sustainability considerations [1].

## 2. Insect-Farming By-Products as Raw Materials for Chitosan Production

The resource requirements of insect farming depend on substrate, production system and processing conditions [9]. The integration of insect farming within circular economy frameworks, particularly through bioconversion of organic waste into valuable biomass, adds substantial environmental and economic value [9]. Potential resource savings during insect rearing do not by themselves establish lower environmental impact or cost for purified chitosan production [9]. Unlike crustacean processing, which depends on seasonal harvesting and geographic limitations, insect farming can operate year-round in controlled environments [10].

For *Hermetia illucens*, primary studies have examined exuviae, puparia, processing flakes, and dead adults collected as farm residues. In the study by [2], the provenance of the puparia and dead adults was documented as insect-farming material; the raw samples differed in composition before extraction. On a dry-mass basis, puparia contained 16.0% minerals, 30.0% protein and 5.0% lipid, whereas the dead-adult stream contained 8.1%, 49.0% and 19.7%, respectively [2,7].

Developmental stage affected measured recovery and purification behaviour. In the same study, bleached chitin yields relative to original raw biomass were 1 ± 0.7% for larvae, 23 ± 1.9% for puparia and 6 ± 0.1% for dead adults [2]. A separate farm-side-stream study reported chitin contents of 21–33% for puparia, 20–28% for processing flakes and 7–13% for adult insects, depending on the analytical method [11]. Across life stages, exuviae were more difficult to purify than other BSF materials, while prepupal and puparium chitin was more crystalline; after a common deacetylation treatment, chitosan reached a degree of deacetylation of 89%, although the exuviae were less reactive [12]. Developmental stage and cuticle structure may affect extraction [13].

Composition can also determine process sequence. Sequential extraction of BSF life stages required lipid removal followed by protein extraction and acid-base chitin purification; protein recovery varied from 27% to 57%, and the purified chitin had an acetylation degree of approximately 90% [14]. Exuviae studies likewise required demineralisation and deproteinisation before deacetylation, with formic acid loading and reaction conditions affecting chitin content and chitosan yield [15]. These findings support adapting delipidation, deproteinisation, demineralisation and bleaching to the stream rather than transferring a crustacean protocol unchanged.

For *Tenebrio molitor*, exuviae, breeding rejects, and processing residues should be considered separately from whole larvae or adults harvested specifically for extraction. In breeding rejects, delipidation was required to remove impurities from exuviae chitin before pure α-chitin was obtained; subsequent deacetylation produced highly deacetylated chitosan with process-dependent molar mass [4]. Adult processing residues subjected to enzyme-assisted extraction contained 0.9–4.8 g/100 g residual lipid, 0–0.5 g/100 g ash and approximately 7% moisture; the resulting chitosan had a degree of deacetylation of 85% [16]. In a whole-body comparison, exuviae gave an 18.01% dry-weight chitin yield, whereas whole body gave 4.92%; the latter is not evidence of a farming residue [17].

Diet and storage require more controlled evidence. BSF puparia in one primary study came from insects reared on plant-origin food-processing by-products and were stored at −18 °C before extraction [7]. The study did not test alternative diets or storage durations, and the available by-product studies do not establish how organic-waste composition, ageing or storage alters subsequent chitin purity, chitosan degree of deacetylation or molecular mass. Extraction protocols should therefore be matched to the documented material stream, its composition and its developmental history, with chitin measurements kept distinct from properties measured only after conversion to chitosan.

## 3. Extraction Routes

Extraction routes for insect-farming by-products are compared here by processing approach: chemical extraction, enzyme-assisted and biological extraction, and alternative-solvent or intensified processes. Within each route, the species and material stream are retained because they affect treatment requirements and recovery. Chitin yields relative to biomass are distinguished from chitosan recoveries relative to isolated chitin, and whole-insect studies are identified as methodological comparators rather than direct evidence of by-product valorisation.

### 3.1. Chemical Extraction

Conventional chemical routes combine acid demineralisation and alkaline deproteinisation with concentrated-alkali deacetylation. For *H. illucens* exuviae, a scaled procedure combined demineralisation of 1 kg of ground material in 10 L of 0.5 M formic acid at room temperature for 1 h with deproteinisation in 2 M NaOH at 80 °C for 2 h. Subsequent heterogeneous deacetylation used 12 M NaOH under nitrogen, with different solid loadings, temperatures and reaction times. Treatments at 140 °C for 3 h, 140 °C for 6 h and 120 °C for 3 h gave chitosan yields of 22%, 38% and 47% relative to chitin, respectively, corresponding to 8%, 13% and 16% of the starting biomass. The homogeneous route involved 10 M NaOH at 4 °C for 12 h, ice addition and separation of the soluble fraction, followed by incubation at room temperature for 48 h. Its recovery was lower, at 13% of chitin and 4% of biomass. Acid dissolution, precipitation and washing formed part of product recovery, so these yields represent the complete procedures rather than the alkaline reaction alone [15]. Bleaching introduces an additional process variable. For *H. illucens* empty puparia and dead adults, one route used 0.5 M formic acid at room temperature for 1 h and 2 M NaOH at 80 °C for 2 h, with bleaching applied to a separate subset using 5% H_2_O_2_ at 90 °C for 30–60 min. Both bleached and unbleached chitin were deacetylated in 12 M NaOH at 100 °C for 4 h; chitosan was then dissolved in 1% acetic acid and precipitated at pH 8. In puparia, bleaching was associated with an increase in chitosan recovery from 28 ± 4.5% to 42 ± 1.5% relative to chitin, and from 8 ± 0.8% to 10 ± 0.2% relative to biomass. For dead adults, the corresponding recoveries increased from 27 ± 2% to 41 ± 1% of chitin and from 2 ± 0.4% to 3 ± 0.3% of biomass [2]. A related protocol applied to both by-products used a 1 h bleaching treatment, the same 12 M NaOH deacetylation conditions, acetic acid purification and lyophilisation, but provided no extraction-yield data [18].

As an alternative to hot heterogeneous treatment, homogeneous deacetylation was investigated for *H. illucens* puparia and dead adults. After formic acid demineralisation, alkaline deproteinisation and optional H_2_O_2_ bleaching, chitin was treated with 12 M NaOH at 4 °C overnight. Ice addition and centrifugation preceded incubation of the supernatant at room temperature for 24–48 h; HCl precipitation and a further acetic acid dissolution-precipitation cycle recovered chitosan. For puparia, unbleached and bleached materials yielded 10.9 ± 2.4% and 33 ± 7.8% relative to chitin, respectively, equivalent to 3 ± 0.8% and 7 ± 2.4% of biomass. Dead-adult material gave 10 ± 2.3% and 30 ± 0.5% relative to chitin, and 1.0 ± 0.4% and 2.0 ± 0.1% relative to biomass [19]. Both homogeneous and heterogeneous routes were also applied to puparia in a further study, although yields were not reported [20].

Alternative alkaline systems include the Broussignac and Kurita processes. A study of *H. illucens* processed exuviae collected at 4–6, 8–12 and 15–20 days. Extraction comprised two 0.55 M HCl baths at room temperature, decolourisation with H_2_O_2_/NaHCO_3_, and four to five 45 min deproteinisation baths in 1 M NaOH at 80 °C. Broussignac deacetylation used a KOH:ethanol:monoethylene glycol mixture (50:25:25) at 120 °C for 24 h. Recovery relative to chitin was 71%, 72% and 73% for the three age groups before purification, decreasing to 55%, 58% and 64% after acetic acid dissolution, filtration and reprecipitation. This loss during purification is relevant when comparing extraction performance. The same study reported 74% recovery from puparia chitin, equivalent to 32% of the starting material, without the additional purification required for exuviae. For dead adults, Broussignac and Kurita deacetylation—the latter using 50% NaOH at 120 °C—gave 74–78% and 69–72% recovery relative to chitin, respectively, and 9.5% and 8.3% relative to biomass. The adult yields were reported as process ranges rather than individual values for each reaction time [13].

The extraction sequence preceding these deacetylation treatments was also examined in *T. molitor* breeding rejects. Two sequences were compared across exuviae and dead adults. P1 combined 0.55 M HCl demineralisation, H_2_O_2_/NaHCO_3_ decolourisation and repeated 1 M NaOH deproteinisation at 80 °C. P2 replaced the acid/bleaching sequence with chloroform-methanol delipidisation followed by the same alkaline deproteinisation. Subsequent deacetylation used either the Broussignac mixture or 50% NaOH according to the Kurita process, at 120 °C. P2 followed by Broussignac treatment gave mean chitosan recoveries of 76% and 75% relative to chitin for exuviae collected at 25–35 days and after 60 days, respectively, corresponding to 20.5% and 21% of raw biomass. For dead adults, Broussignac treatment yielded means of 78% after P1 and 80% after P2, equivalent to 19.5% and 24% of biomass. These grouped recoveries should not be assigned to an individual deacetylation time [4].

Other conventional protocols for *T. molitor* exuviae used 2 N HCl at 20 °C for 3 h and 5% NaOH at 95 °C for 3 h, followed by deacetylation in 55% NaOH at 105 °C for 5 h [17]. A related procedure used 2 M HCl at room temperature, the same 5% NaOH deproteinisation conditions, and 50% NaOH at 105 °C for 5 h. It yielded 7.41 ± 0.26% chitosan relative to dry exuviae and 57.76 ± 3.68% relative to chitin. Acid dissolution and NaOH reprecipitation were applied to a separate aliquot for additional purification [21].

A further procedure applied to mealworm exuviae and dead adults used 1 M HCl at 60 °C for 1.5 h, 1 M NaOH at 100 °C for 20 h, and decolourisation in water: 95% ethanol:chloroform (4:2:1) for 2 h. Deacetylation required 60% NaOH at 100 °C for 20 h. The recorded chitin yields were 13.25 ± 0.63% for exuviae and 15.13 ± 0.78% for dead adults, relative to insect mass. The corresponding chitosan yields were 6.26 ± 0.10% and 6.52 ± 0.06% relative to insect dry mass; and 74.93 ± 0.93% and 78.96 ± 0.45% relative to dry chitin [5]. The two yield bases are retained as reported and should not be pooled or treated as an independently validated mass balance.

Further chemical protocols for *H. illucens* puparia differed substantially in treatment duration and reagent choice. One combined 3 M HCl and 1 M NaOH treatments, each for 1 h at 25 °C, with 12% NaOCl depigmentation for 10 min and deacetylation in 50% NaOH at 80 °C for 1 h. It reported 50.83% chitin recovery from dry exuviae and 36% chitosan recovery from chitin [22]. A cocoon/dead-fly comparison instead used 5% NaOH, 4% HCl and 1% KMnO_4_ for deproteinisation, demineralisation and decolourisation, respectively. Chitosan recovery was 27.64% from puparia and 4.06% from dead flies, relative to the original biomass; the corresponding chitin yields were 37.03% and 9.72%. Deproteinisation used 90 °C for 4 h, demineralisation and decolourisation each proceeded at room temperature for 12 h, and deacetylation used 70% NaOH at 80 °C for 8 h [23]. A more prolonged pupal-exoskeleton route used 1 M HCl at room temperature for 1 h, thirteen 1 h cycles of 1 M NaOH at 80 °C, and deacetylation in 50% NaOH at 90 °C for 10 h. Chitin recovery was reported as 29%, whereas the subsequent chitosan yield was not reported [24].

Whole-insect studies provide additional methodological options for future testing on residual streams. In *H. illucens* prepupae, 0.5 M formic acid treatment for 30 min and 2 M NaOH at 80 °C for 2 h were followed by deacetylation in 12 M NaOH at 90 °C for 3 h. Mean chitosan yields relative to dry biomass were 4.69%, 3.39%, 3.30%, 2.97% and 2.40% for standard, fruit, meat, vegetable and mixed diets, respectively; the corresponding chitin-based recoveries were 67.22%, 65.24%, 68.05%, 64.64% and 64.91%. Diet-related differences in chitosan recovery were not significant. Bleaching was performed after deacetylation, using 5% H_2_O_2_ at 90 °C for 15 min, and retained only approximately 13–19% of the unbleached chitosan. This post-deacetylation bleaching strategy was demonstrated in prepupae, not in a by-product stream, but provides a basis for testing how bleaching order affects recovery from insect residues [25].

Similarly, adult *T. molitor* used as experimental biomass was processed by two routes. The first combined 2 M HCl at 65–75 °C for 2 h, 2 M NaOH at 80–90 °C for 16 h, and chloroform:methanol:water decolourisation. The second used repeated 3% NaClO treatment at 100 °C for 10 min, 1 M HCl at 75 °C for 15 min and 1 M NaOH at 100 °C for 20 min. Both employed 50% NaOH at 100 °C for 3 h for deacetylation. Chitin yields were 13.3% and 17.7% of dry beetle mass, while chitosan recovery from chitin was 78.26% and 76.43%, respectively [26]. These procedures may guide future adult-residue processing, but their application to harvested beetles does not establish performance in farming by-products. The homogeneous and heterogeneous BSF protocols described above were also applied to adults classified as non-by-products, without reported yields; these adult results likewise provide methodological guidance rather than direct evidence of residue valorisation [20].

### 3.2. Enzymatic and Biological Extraction

The biological approaches documented for mealworm by-products combine enzymatic protein removal with subsequent chemical deacetylation. For larval cuticles/exuviae, Alcalase was applied at 2% enzyme/substrate to a 3% dispersion at pH 8 and 50 °C, with hydrolysis continued until a plateau. The enzyme was then inactivated at 90 °C for 15 min, and the solid fraction was recovered by centrifugation. Demineralisation was omitted, and the recovered chitin was deacetylated using 40% NaOH at 90 °C for 8 h under stirring. Reported chitosan recovery was 45.1% relative to chitin, with an overall cuticle-to-chitosan yield of 31.9% [27]. The enzymatic stage therefore replaced alkaline deproteinisation, while chitosan production still required concentrated alkali.

Enzyme-assisted extraction was also investigated for adult mealworm processing residues. Ground and sieved material underwent deproteinisation optimised through a face-centred central composite design varying pH, enzyme concentration and treatment time. The selected enzymatic treatment used 2% Alcalase at pH 10 and 70 °C for 6 h. Subsequent deacetylation used 60% NaOH at a 1:10 solid-to-liquid ratio, 100 °C for 24 h, followed by 30% H_2_O_2_ treatment at 90 °C for 30 min; chitosan recovery was not reported [16]. These studies establish enzymatic pretreatment as an option for both exuviae and adult residues; they do not provide a directly comparable yield-based assessment across the two streams.

### 3.3. Alternative-Solvent and Intensified Processes

Autoclave-assisted deacetylation was evaluated alongside changes in the extraction sequence for *T. molitor* exuviae. Method M1 used 5% HCl at 40 °C for 2 h, 3% NaOH at 60 °C for 4 h, and 50% NaOH at 80 °C for 2 h for deacetylation. M2 omitted the acid step while retaining the other treatments. Their chitosan yields were 6.77 ± 0.38% and 8.27 ± 0.60% of dry biomass, respectively. M3 combined 5% NaOH deproteinisation at 70 °C for 3.5 h with pressurised deacetylation in 50% NaOH at 121 °C and 15 psi for 30 min, giving 8.87 ± 0.35%. Thus, the highest recovery in this comparison followed a shorter, pressurised deacetylation step and an extraction sequence without acid demineralisation [28].

Autoclave-assisted processing was also compared with conventional heating for *H. illucens* pupal cases. Four routes differed in delipidisation and subsequent treatment: P1 omitted delipidisation, P2 used hexane/Soxhlet extraction, and P3/P4 used methanol:chloroform (1:4, *v*/*v*). All included 0.55 M HCl demineralisation. P1-P3 used 1 M NaOH at 80 °C for 18 h and Broussignac deacetylation at 120 °C for 24 h. In P4, deproteinisation comprised five 15 min autoclave treatments at 121 °C and 2.2 bar, followed by deacetylation under the same temperature and pressure for 1 h. Chitosan recoveries relative to chitin were 68.57 ± 1.15%, 72.02 ± 0.57%, 74.37 ± 0.66% and 83.33 ± 1.15% for P1-P4, respectively. Within this comparison, autoclave processing combined shorter alkaline treatments with the highest recovery [29].

Microwave assistance was applied to BSF breeding residues described by the authors as “prepupal cases”, whose correspondence to exuviae or post-emergence puparia is not explicitly resolved. At 600 W, methanol:chloroform delipidisation required 2 min, 0.55 M HCl demineralisation 4 min, and 1 M NaOH deproteinisation five 6 min baths. Microwave-assisted Broussignac deacetylation then required 20 min. Chitin recovery was 21.14% of initial biomass and chitosan recovery was 65.89% of chitin [30]. For exuviae, another approach replaced conventional protein hydrolysis with superheated water at 150 °C for up to 20 h, after defatting and demineralisation. It recovered 20% purified chitin from exuviae; subsequent NaOH deacetylation was recorded, but its detailed conditions and chitosan yield were unavailable [31].

Additional evidence for microwave-assisted extraction comes from BSF pupae classified as non-by-product biomass. The conventional sequence used 3 M HCl at 50 °C for 2 h, 2 M NaOH at 80 °C for 2 h, and 12 M NaOH at 100 °C for 2 h, followed by acid dissolution and alkaline precipitation. Applying 800 W microwave irradiation to the three stages reduced the combined optimised treatment time to 24 min. Chitosan yields relative to starting biomass were 3.28 ± 0.35% with conventional heating and 3.89 ± 2.35% with microwave assistance [32]. These results guide future testing on puparia or other residues; the reported yield difference alone does not establish a recovery advantage for by-product processing.

A deep eutectic solvent route used choline chloride:lactic acid (1:2 molar) to extract chitin from BSF pupal shells after petroleum-ether defatting. Optimised extraction conditions were 92 °C for 5.6 h at 20 mL g^−1^, followed by washing and H_2_O_2_ bleaching. The recorded yield of 28.40% concerns precursor chitin. Subsequent partial deacetylation used NaOH at a solid:liquid ratio of 1:30, with 33% NaOH at 90 °C for 3 h for Cts1/Cts2 and 42% NaOH at 120 °C for 4.5 h for Cts3. Cts1 used a 60-mesh precursor and Cts2/Cts3 an 80-mesh precursor; chitosan yields were not reported [33]. This route consequently supports discussion of alternative chitin extraction, while its complete recovery to chitosan cannot be compared quantitatively with the conventional routes.

Microwave treatment must also be distinguished from post-extraction depolymerisation. In a study using adult mealworms classified as non-by-products, extraction involved 2 M HCl and 2 M NaOH treatments at 50 °C for 24 h each, solvent purification, and deacetylation in 60% NaOH at 120 °C for 2 h. Chitin recovery was 12.49 ± 0.27% of insect mass, whereas chitosan recovery was not recorded. Microwave irradiation at 800 W for 6, 18 or 30 min was subsequently applied to chitosan solutions in acetic acid [34]. This is a possible downstream treatment for future by-product-derived chitosan, rather than evidence of microwave-assisted extraction from residues.

Across these studies, the strongest comparisons are those made within the same biomass and analytical framework. Bleaching can increase recovery from isolated chitin while reducing precursor recovery, and additional purification can remove mass that would otherwise be counted as product. Enzyme-assisted routes replace particular purification steps but commonly retain concentrated-alkali deacetylation. Shorter microwave or autoclave treatments therefore warrant evaluation through complete mass and resource balances, rather than treatment duration alone. Evidence for ionic-liquid integration in other insect materials is discussed in Section 7.3; no directly comparable mechanochemical route for the target by-products is established by the studies reviewed here.

## 4. Chemical Structure and Physicochemical Characterisation of Insect-Derived Chitosan

The utilisation of insect production by-products as a source of chitin to produce chitosan represents a promising avenue for sustainable biopolymer development. However, ensuring that chitosan derived from these alternative sources meets established quality standards and possesses characteristics comparable to conventionally produced chitosan is essential for widespread industrial and commercial adoption.

### 4.1. Deacetylation Degree and Molecular Weight

The characterisation of insect-derived chitosan must ensure multiple physicochemical parameters that define its functionality and performance. These parameters include molecular weight (average and/or distribution), degree of deacetylation (DDA), crystallinity and purity analysis [35].

Among the properties affected by the extraction and deacetylation conditions described in Section 3, the degree of deacetylation (DDA) provides a measure of the extent of deacetylation. A higher DDA corresponds to a greater proportion of free amino groups (-NH_2_), which can participate in interactions with biological molecules and contribute to chitosan’s functional properties and bioactivity [36].

Several analytical techniques are employed to determine DDA, each offering distinct advantages. FTIR spectroscopy (Figure 1) is a rapid, non-destructive method for identifying functional groups and assessing the DDA [37], and the consistency of its characteristic absorption bands across insect- and crustacean-derived samples supports their structural equivalence [2]. The DDA can be calculated directly from band intensity ratios [38].

Titration is one of the simplest, most cost-effective and most accessible methods [39]. It relies on the neutralisation of protonated amino groups [40] by a standardised base such as NaOH [41], and the DDA is calculated from the net volume of base consumed [42]. Potentiometric detection is the most recommended approach, as it provides precise endpoint determination and avoids the subjectivity of visual indicators [43], which are less reliable because chitosan has low solubility at neutral to basic pH [44].

Finally, nuclear magnetic resonance (NMR) is one of the most powerful and precise techniques for determining the DDA [45] and is regarded as the gold standard for validating other methods [46]. ^1^H NMR (Figure 2) distinguishes acetylated from deacetylated units and allows their relative proportions to be quantified [42], while ^13^C NMR provides complementary information on the pattern of acetylation and molecular organisation [46,47]. The DDA is obtained from the integration of the corresponding peak areas [42,46]. Although NMR requires sophisticated instrumentation and trained personnel [46], it should be used periodically to verify and calibrate faster methods such as FTIR and titration, which are preferable for routine quality control [46].

DDA should be considered alongside molecular weight (MW) and its distribution, as chitosan samples consist of polymer chains of different lengths. MW is expressed in daltons (Da) or kilodaltons (kDa) and may be reported as number-average (M_n_), weight-average (M*w*) or viscosity-average (M_v_) molecular weight. These averages describe different aspects of the polymer population and are therefore not interchangeable. MW can be determined using different techniques, with the choice depending on the sample characteristics and the information required [48].

Viscometry is one of the simplest, most cost-effective and accessible approaches, requiring no expensive instrumentation and being suitable for both crude extracts and purified chitosan [49]. Intrinsic viscosity is converted into M_v_ using the Mark–Houwink–Sakurada equation [49], whose constants depend on factors including DDA, pH, ionic strength and solvent composition [50].

SEC/GPC is a more versatile approach because it separates polymer chains according to size and provides the molecular weight distribution rather than a single average value [51]. When refractive index (RI) detection is combined with calibration standards, however, the method assumes that chitosan behaves similarly to the standards, which is not always the case [52]. Coupling size-exclusion chromatography with multi-angle light scattering and RI detection (SEC-MALS-RI) overcomes this limitation by providing absolute M*w* and molecular weight distribution without external calibration standards, while also allowing aggregate signals to be distinguished [52,53]. Static light scattering can likewise determine absolute M*w* without calibration curves by measuring scattered light intensity at several concentrations and extrapolating to zero concentration [54].

MALDI-TOF MS, in contrast, directly measures the mass of individual molecules and is particularly useful for chitosan oligosaccharides and low-molecular-weight fractions, where individual oligomers and their deacetylation patterns can be resolved [55]. Although it may also be applied to higher-molecular-weight polymers, adequate sample dissolution is essential [56].

In practice, viscometry provides a favourable balance between cost, speed and accuracy for routine characterisation of higher-molecular-weight chitosan, whereas SEC/GPC is more informative when the molecular weight distribution is required. Light-scattering approaches are particularly valuable for absolute MW determination, while MALDI-TOF MS is better suited to low-molecular-weight species [48].

For insect-derived chitosan, viscometry and SEC/GPC can therefore serve as primary methods, as established viscometric relationships and SEC/GPC procedures can provide results comparable with those obtained for crustacean chitosan when samples are processed under similar conditions [57].

For interpreting these differences, the molecular-weight classification used here distinguishes high-molecular-weight chitosan (HMWC, >700 kDa), medium-molecular-weight chitosan (MMWC, 150–700 kDa) and low-molecular-weight chitosan (LMWC, <150 kDa). These categories provide a framework for discussing the physicochemical and biological properties of the extracted materials and their suitability for different applications [58].

Considered together, DDA, molecular weight and molecular-weight distribution help interpret differences in chitosan solubility, viscosity and biological performance, including antimicrobial activity and biocompatibility [57].

The importance of considering DDA and viscosity separately is illustrated by BSF exuviae, for which the heterogeneous treatments described in Section 3 produced potentiometric DDA values of 44% at 140 °C for 3 h, 72% at 140 °C for 6 h and 43% at 120 °C for 3 h. The homogeneous treatment, comprising cold alkaline pretreatment followed by reaction at room temperature, gave 34%. Molecular weight was not reported. Dynamic viscosities were 31, 77 and 277 mPa·s for the three heterogeneous products and 509 mPa·s for the homogeneous product; these solution-viscosity measurements cannot substitute for molecular-weight values [15]. The comparison separates the highest DDA, obtained with the longer 140 °C treatment, from the highest measured viscosity.

A further comparison using Broussignac treatment of BSF exuviae at 120 °C for 24 h gave different results across collection ages. For LE1, LE2 and LE3, collected at 4–6, 8–12 and 15–20 days, DDA calculated as 100—DA from ^1^H NMR was 98%, 100% and 100%, respectively. The corresponding potentiometric values for soluble fractions were 93%, 95% and 96%. M_v_ were 95, 101 and 110 kDa; the separately analysed soluble fractions gave 94, 100 and 109 kDa. Under the same Broussignac framework, puparia chitosan had 96% DDA by NMR, 91% by titration and M_v_ of 115 kDa. For dead adults, extending Broussignac treatment from 4 to 24 h increased NMR-derived DDA from 91% to 97% and reduced M_v_ from 188 to 130 kDa. Kurita treatment of adult material gave 97% DDA after 24 h but M_v_ of 32 kDa, demonstrating that similar DDA values can accompany substantially different chain-size measurements [13].

Bleaching also distinguished products from BSF puparia and dead adults. After heterogeneous deacetylation in 12 M NaOH at 100 °C for 4 h, unbleached puparia chitosan had a titration-derived DDA of 83 ± 3.1% and viscometric molecular mass of 55 ± 1.7 kDa, compared with 90 ± 4.2% and 35 ± 4.3 kDa after bleaching. Dead-adult products showed the same direction of change: 91 ± 0.7% and 62 ± 0.1 kDa without bleaching, versus 93 ± 1.4% and 36 ± 0.5 kDa with bleaching [2]. Under homogeneous deacetylation, puparia products instead gave 62 ± 3.5% DDA and 285 ± 29 kDa without decolourisation, and 72 ± 3.6% and 115 ± 16 kDa after decolourisation. The corresponding dead-adult values were 59 ± 2.9% and 258 ± 28 kDa, and 61 ± 2.3% and 89 ± 6 kDa [19]. These are separate experimental comparisons; they support treatment-specific differences rather than a universal bleaching response.

Other puparial routes broaden the observed range. The short chemical protocol using NaOCl depigmentation and 50% NaOH deacetylation at 80 °C produced chitosan with 53% DDA by FTIR [22]. The NaOH/HCl/KMnO_4_ route gave DDA values of 72.3% for cocoon-derived material and 71.9% for dead-fly material, with viscometric molecular masses of 194.9 and 164.6 kDa, respectively [23]. For the P1-P4 pupal-case comparison, NMR-derived DDA values were 94.96%, 95.23%, 96.15% and 97.08%, while M_v_ values were 166.964, 95.316, 109.874 and 220.378 kDa. The autoclave route P4 therefore combined the highest DDA and M_v_ within that experiment. FTIR gave lower DDA values of 90.91%, 93.67%, 93.65% and 94.20%, illustrating why the analytical method must accompany the result [29]. Microwave-assisted extraction of residues described as prepupal cases gave 97.7% DDA, calculated from 2.3% DA by ^1^H NMR, and a reported molecular mass of 155 kDa [30].

DES-derived pupal-shell products require separate terminology. The author-labelled Cts1, Cts2 and Cts3 series had DDA values of 36.3%, 41.85% and 65.3%, respectively, and should be presented as partially deacetylated materials. Individual molecular masses were unavailable, although the series was reported to be of the order of 10^5^ g mol^−1^ [33]. Likewise, bulk chitosan from puparia and its STPP-based nanochitosan formulation are distinct products: their FTIR-derived DDA values were 62.3% and 85.3%, respectively [24]. This difference does not, by itself, establish that nanoparticle formation caused further chemical deacetylation.

Similar processing-dependent differences were observed for mealworm exuviae: the P2 extraction route followed by Broussignac treatment showed a time-dependent DDA- M_v_ relationship. In LE1, collected at 25–35 days, increasing deacetylation from 6 to 24 h changed NMR-derived DDA from 88.5% to 94% and M_v_ from 284 to 136 kDa; in LE2, collected after 60 days, the corresponding changes were 89% to 92% and 339 to 148 kDa. After P1 and 24 h of Broussignac treatment, LE1 and LE2 gave 95% and 86% DDA and M_v_ values of 83 and 96 kDa. For dead adults, P2/Broussignac treatment from 4 to 24 h increased DDA from 91.5% to 95% while reducing M_v_ from 284 to 153 kDa. The recorded P1/Broussignac adult DDA at 24 h is internally inconsistent and is therefore excluded from this comparison [4].

The M1-M3 mealworm-exuviae comparison further demonstrates that extraction sequence matters. M1, comprising acid demineralisation and conventional alkaline treatment, gave 42.62 ± 0.38% DDA and M_v_ of 71 ± 0.05 kDa. Omitting demineralisation in M2 gave 25.25 ± 1.2% and 97 ± 1.17 kDa. M3, using pressurised deacetylation at 121 °C, gave 55.62 ± 0.79% and 116 ± 2.53 kDa [28]. Another exuviae protocol using 55% NaOH at 105 °C for 5 h gave 50.38% DDA by titration [28], while treatment with 50% NaOH under the same temperature and duration in a separate study gave 72.27 ± 1.03% DDA and M_v_ of 612 kDa [21]. For the prolonged 60% NaOH route, mealworm exuviae and dead adults had titration-derived DDA values of 76.32 ± 0.26% and 89.21 ± 0.96%, respectively; FTIR estimates were 81.06% and 87.29% [5]. Enzymatic pretreatment followed by chemical deacetylation gave 53.9% DDA for larval cuticles/exuviae [27] and a reported 85% for adult processing residues [16].

The non-by-product studies discussed in Section 3 provide additional, explicitly provisional guidance. In BSF pupae, conventional and microwave-assisted routes gave similar FTIR-derived DDA values of 76.48% and 76.01%, respectively [32]. In harvested adult mealworms, the two chemical protocols described there gave DDA values of 75.84% and 72.6% [26]. Neither experiment establishes the same values for residual streams. For BSF prepupae, the post-deacetylation bleaching route was characterised by ATR-FTIR relative acetylation content (RAC), not quantitative DDA. Unbleached RAC depended on diet, whereas bleached products converged towards approximately 0.3 without significant diet differences [25]. This supports testing whether the treatment reduces feedstock-related spectral differences in by-products but does not justify predicting their DDA or molecular weight.

The adult-mealworm microwave depolymerisation study, also identified as non-by-product evidence in Section 3, reported molecular-weight values of 492.49 ± 45.37 kDa for native chitosan, 419.02 ± 23.40 kDa for the freeze-dried control, and 378.17 ± 9.32, 311.26 ± 18.49 and 328.90 ± 2.72 kDa after 6, 18 and 30 min, respectively. DDA remained within approximately 50–52% across these samples [34]. This suggests a testable possibility of reducing chain size without a large DDA change in future by-product-derived chitosan.

### 4.2. Crystallinity, Thermal Stability, and Morphology

Beyond DDA and molecular weight, crystallinity, morphology and thermal behaviour provide complementary information on how extraction and subsequent treatment affect chitosan structure and performance [2].

Crystallinity describes the organisation of ordered and amorphous regions within the polymer, and X-ray diffraction (XRD; Figure 3) is the primary and most widely accepted technique for determining it [59]. It directs X-rays at the sample and measures the diffraction patterns produced by the ordered molecular structure, with sharp, intense peaks indicating crystalline regions and broader, less intense signals indicating amorphous regions, which enables the crystallinity index to be quantified [24,59,60]. Chitosan shows a distinctive peak at 2θ ≈ 10°, arising from the repeating d-spacing within ordered crystalline domains [61], while the main crystalline peak at 2θ = 20° serves as the reference intensity for the calculation [62].

Thermogravimetric analysis (TGA; Figure 4) is a thermal characterisation technique that measures mass loss as a function of temperature during controlled heating [63]. This technique is primarily used for assessing thermal stability and degradation kinetics [64,65].

Scanning electron microscopy (SEM) provides high-resolution images of chitosan surface morphology, including texture, porosity and fibrillar organisation, revealing structural features that correlate with the degree of molecular organisation [66]. These features may reflect processing history [2].

The bleaching comparisons discussed in Section 3.1 also reveal changes in these structural properties. For heterogeneously deacetylated BSF chitosan, bleaching increased the reported crystallinity index (CrI) from 78% to 80% in puparia products and from 79% to 86% in dead-adult products. Unbleached puparia chitosan had a rough, porous and more visibly fibrillated surface; bleaching gave a more homogeneous, less fibrillated material. Both formed uniform films, with improved optical clarity after bleaching. Dead-adult films remained darker: bleaching lightened them, but residual pigmentation persisted [2]. Homogeneously deacetylated puparia products had CrI values of 51% without decolourisation and 68% after decolourisation, accompanied by fewer visible particulate impurities in the latter. Decolourised adult products were described as smoother, with less pronounced filamentous structures; their numerical CrI values are not used because the record assigns an incompatible unit [19].

For BSF breeding rejects processed by the Broussignac route, SEM observations identified porous chitosan from LE1 exuviae and puparia, and a retained fibrous morphology in adult-derived chitosan (Figure 5) [13]. In the intensified pupal-case comparison, P1-P4 chitosans had CrI values of 34.8%, 36.23%, 45.3% and 47.7%, respectively, and DTG maxima of 323, 338, 343 and 345 °C. All showed heterogeneous surfaces with disorganised microfibrils, with lighter pores particularly evident in P2/P3 [29]. Microwave-extracted prepupal-case chitosan had CrI of 51.26% and a DTG maximum of 326 °C [30], whereas the short NaOCl-based puparial route gave CrI of 57% [22]. These chitosan values should be compared within their processing frameworks rather than combined with crystallinity measurements of precursor chitin.

Bulk chitosan from puparia had a CrI of 77.2% and a rough honeycomb-like surface (Figure 4) with fibres and large pores. Its nanochitosan formulation had a lower CrI of 71.4% and dense particulate or lamellar aggregates. TEM gave a mean particle size of 34.05 ± 10.24 nm, whereas DLS gave a hydrodynamic size of approximately 300 nm; these measurements describe different particle-size observables. The main DTG maxima were 316.9 °C for bulk chitosan and 325.4 °C for nanochitosan, with residual masses at 600 °C of 14.7% and 17.7%, respectively [24]. In the DES-derived Cts series, crystallinity decreased with increasing deacetylation, but numerical indices were unavailable. Particles remained larger than 100 µm, and all three partially deacetylated products had positive zeta potentials, with Cts3 highest [33].

For mealworm residues subjected to the prolonged chemical route, exuviae-derived chitosan had a reported crystallinity index of 65.3%, DTG maximum of 245.8 °C and thermal residue of 4.14%; adult-derived chitosan gave 67.6%, 326.8 °C and 30.91%, respectively. SEM described a rough exuviae-derived surface and pores in the adult-derived material [5]. A separate exuviae study showed that additional purification changed a rough, flaky and compact chitosan into a smoother, porous and fibrous material (Figure 6). Its unpurified product had a DTGmax of 306.69 °C; DSC peaks shifted from 155.0 and 312.5 °C before purification to 127.1 and 287.7 °C afterwards [21]. Enzymatically pretreated exuviae chitosan showed decomposition events at 273–321 °C [27], while the adult-residue product had porous microfibrillar morphology and major thermal degradation above 230 °C [16].

For non-by-product BSF pupae, the conventional and microwave routes already described in Section 3 gave CrI values of 37.76% and 20.33%, respectively. Conventional processing produced a denser, more compact surface, whereas microwave processing produced a more porous and microfibrillar material [32]. This suggests that microwave processing could be tested to alter structural organisation in puparial chitosan, but the direction and magnitude require confirmation in that residual stream. In the non-by-product adult-mealworm study, native, freeze-dried-control and 6-, 18- and 30 min irradiated chitosans had CrI values of 73.84%, 70.10%, 71.09%, 66.26% and 69.10%, respectively. Freeze-drying introduced more pits and cracks, while irradiation caused smaller additional morphological changes. DTG maxima remained close to 297–302 °C [34]. These observations indicate why drying controls should accompany future irradiation experiments on by-product-derived chitosan.

### 4.3. Purification Efficiency and Residual Components

The processing-related differences in DDA, molecular weight and structure described above must also be considered alongside residual-component measurements. These measurements complement polymer characterisation by assessing chitosan purity and quality for industrial and biomedical applications [67].

This distinction is particularly important when evaluating purification: residual components must be measured in the final chitosan rather than inferred from the precursor chitin. BSF larval-exuviae products obtained by Broussignac treatment contained 10%, 6% and 3% insoluble impurities for LE1, LE2 and LE3, respectively, before additional purification. Acid dissolution, filtration and reprecipitation removed these fractions, and LE1 SEM showed a more organised porous surface after purification. The impurity percentages describe insoluble material and should not be relabelled as residual protein or ash. Puparia and adult-derived products in the same study did not require this additional purification [13]. For mealworm exuviae, P1/Broussignac products retained organic impurities, whereas P2, incorporating delipidisation, produced material described as pure and soluble by 6 h. Both P1 and P2 adult products were described as pure, but quantitative residual-component measurements were not recorded [4].

Measured composition varied across BSF puparial routes. The short NaOCl-based protocol gave chitosan with 4.41% moisture and 7.45% ash [22]. In the cocoon/dead-fly comparison, residual protein and ash were 0.29% and 0.03% for cocoon chitosan, versus 0.55% and 0.47% for dead-fly chitosan; solubilities were 93.2% and 94.1%, respectively [23]. Bleaching-related improvements in colour or visible surface cleanliness [2,19] should be distinguished from these quantitative composition measurements.

For mealworm exuviae, chemically extracted chitosan contained 7.66 ± 0.54% moisture and 0.33 ± 0.21% ash; the reported protein estimate of 6.59 ± 0.89% was derived from nitrogen and should retain that methodological qualification [21]. Enzymatically pretreated cuticle/exuviae chitosan contained 4.2 ± 0.1% moisture, 3.7 ± 0.1% ash and 5.4 ± 0.2% protein, with solubility of 40.3 ± 0.6% [27]. The adult-residue enzymatic study recorded approximately 2% residual material as ash in its TGA-based characterisation; this should remain identified by its analytical origin [16]. Thermal residues such as the 14.7% and 17.7% reported for bulk and nanochitosan [24] are likewise retained as thermal-analysis results, rather than treated as interchangeable with separate compositional assays.

Several records provide insufficient final-product composition data. In the DES study, the reported purity of 96.93% belongs to extracted chitin and cannot be assigned to Cts1-Cts3 [33]. The superheated-water study describes thermal behaviour resembling commercial chitosan and SEM of chitosan-treated polyester, without numerical chitosan purity or isolated-polymer morphology [31]. The antimicrobial studies likewise provide little or no quantitative DDA, molecular-weight or residual-component information for the relevant products [18,20]. Such gaps limit comparisons between routes even when chitosan production or application has been demonstrated.

For non-by-product biomass, the two adult-mealworm chemical routes discussed in Section 3 reported solubilities of 95.3 ± 0.4% and 92.3 ± 0.2% [26]. Together with the reduced diet-related RAC differences after bleaching BSF prepupal chitosan [25], these findings identify properties worth measuring when adapting the methods to residues. They support provisional expectations about solubility or spectral consistency, but provide no numerical prediction of residual protein, ash or purity in treated by-products.

Table 1 summarises the extraction conditions, yields and physicochemical properties of chitosan obtained from *H. illucens* and *T. molitor* by-products, with non-by-product materials clearly identified for comparison.

## 5. Chitosan Bioactivity

Chitosan possesses multiple bioactive properties that support its effectiveness across diverse applications. These properties are material- and assay-dependent and result from its molecular structure, specifically amino groups, which become positively charged upon protonation (-NH_3_^+^), and hydroxyl groups that enable interactions with biological systems and pathogens. Amino-group protonation, molecular weight, degree of deacetylation, residual proteins, pH, counter-ions and formulation can all affect the measured response. Direct evidence from *H. illucens* and *T. molitor* is still concentrated in in vitro antimicrobial, epithelial gene-expression and antioxidant assays rather than veterinary clinical or field studies.

### 5.1. Antimicrobial Activity and Mechanisms

Direct evidence from *Hermetia illucens* puparia and dead adults demonstrates why inhibition zones, minimum inhibitory concentrations (MICs) and minimum bactericidal concentrations (MBCs) must be considered separately. After formic acid demineralisation and alkaline deproteinisation, bleached and unbleached chitin were deacetylated with 12 M NaOH at 100 °C for 4 h. Chitosan stocks contained 5 mg/mL in 1% acetic acid; assays tested 1.25, 0.6, 0.3 and 0.15 mg/mL for 24 h at 37 °C, with commercial crustacean chitosan, matched acetic acid solutions, water and bacterial controls [18].

For puparia chitosan, *Escherichia coli* inhibition zones were approximately 9 mm at 0.3–1.25 mg/mL and 8 mm at 0.15 mg/mL for both bleaching conditions. Against *Micrococcus flavus*, bleached material gave 10 ± 0.4 mm at 1.25 mg/mL and 7 ± 0.4 mm at 0.15 mg/mL, compared with 9 ± 0.8 and 7 ± 0.8 mm for unbleached material. MICs for bleached/unbleached puparia products were 0.3/0.15 mg/mL against *E. coli* and 0.15/0.3 mg/mL against *M. flavus*. Dead-adult products generally gave 10 mm zones at 0.3–1.25 mg/mL and 8 mm zones at 0.15 mg/mL. Bleached adult chitosan had MICs of 0.3 and 0.15 mg/mL against *E. coli* and *M. flavus*, respectively; unbleached adult MICs were not determined because solvent inhibition complicated interpretation. Thus, larger adult-derived inhibition zones did not establish uniformly lower MICs, and the near-constant zones at the three higher concentrations did not indicate a proportional dose response. Final assay pH, strain identifiers and numerical commercial-comparator results were unavailable; no MBC should be inferred from these findings [18].

Homogeneous deacetylation produced a different comparison. Following similar acid/alkali extraction with or without H_2_O_2_ bleaching, cold 12 M NaOH treatment was followed by 24–48 h at room temperature and acid dissolution/reprecipitation. Unbleached and bleached puparia products had DDA values of 62 ± 3.5% and 72 ± 3.6%, and MW values of 285 ± 29 and 115 ± 16 kDa, respectively. Dead-adult values were 59 ± 2.9% and 61 ± 2.3% DDA, and 258 ± 28 and 89 ± 6 kDa. Solutions prepared in 1% acetic acid were tested by microdilution at 1.25–0.15 mg/mL for 24 h at 37 °C, with solvent and commercial-chitosan controls. Both puparia products had an *E. coli* MIC of 0.15 mg/mL, whereas bleaching reduced the *M. flavus* MIC from 0.3 to 0.15 mg/mL. Both adult products had MICs of 0.15 mg/mL against both organisms. These results provide no evidence that the higher DDA or lower MW consistently improved inhibition; values at the lowest tested concentration also do not resolve whether lower concentrations would remain effective. Numerical inhibition zone, MBC and comparator outcomes were unavailable [19].

A separate chemical route used 5% NaOH deproteinisation, 4% HCl demineralisation and 1% KMnO_4_ decolourisation before deacetylation. Cocoon-derived chitosan had 72.3% DDA and a viscometric MW of 194.9 kDa; dead-fly chitosan had 71.9% and 164.6 kDa. Agar diffusion at 0.25–1.00% gave *Bacillus subtilis* zones of 4.67–6.33 mm for cocoon material and 4.33–6.00 mm for dead-fly material; both gave 2.34–4.40 mm against *Vibrio alginolyticus* at 1–2%. Tetracycline (30 µg) and 1% acetic acid were controls. The available ranges do not map every concentration to a response, and assay pH, exposure time and control-zone values were unavailable. These are diffusion results, not MIC or MBC measurements [23].

Nanoparticle evidence requires a formulation-specific interpretation. Pupal-exoskeleton chitosan obtained by repeated alkaline deproteinisation and 50% NaOH deacetylation at 90 °C for 10 h was formulated with sodium tripolyphosphate by ionic gelation, sonication and lyophilisation. FTIR-derived DDA was 62.3% for the parent polymer and 85.3% for nanochitosan; MW was unavailable. Only nanochitosan underwent antimicrobial testing. A 5 mg/mL stock in 1% acetic acid was used for agar diffusion and microdilution, with acetic acid and water controls. After 24 h at 37 °C, MIC/MBC values were 160/320 µg/mL against *B. subtilis* and 320/640 µg/mL against *Enterobacter cloacae*. Across 80–1280 µg/mL, zones increased from 6.1 ± 0.1 to 12.4 ± 0.3 mm for *B. subtilis* and from 6.1 ± 0.2 to 13.1 ± 0.3 mm for *E. cloacae*, with relatively little further increase between 640 and 1280 µg/mL. This is direct concentration-dependent evidence for the nanoparticle formulation, but the absence of a tested bulk-polymer comparator prevents attribution of any improvement to nanoparticle formation or chitosan alone [24].

Another puparia study compared homogeneous and heterogeneous deacetylation, each with and without bleaching. Heterogeneous processing used 12 M NaOH at 100 °C for 4 h; homogeneous processing combined cold alkaline treatment with reaction at room temperature. DDA and MW were unavailable. Solutions were tested at 0.5–0.0015 mg/mL by turbidity, with colony counting at 0.5 mg/mL after plating and incubation for 24 h at 37 °C. Unbleached homogeneous puparia chitosan gave 2 × 10^9^ CFU/mL of *Enterococcus faecalis*, compared with 1 × 10^11^ CFU/mL for both bacterial and commercial-chitosan controls; corresponding counts were 1.3 × 10^8^ CFU/mL for *Staphylococcus epidermidis* and 3.3 × 10^8^ CFU/mL for *Streptococcus agalactiae*. The *S. epidermidis* count was reported as three orders of magnitude below the control. Bleached homogeneous and heterogeneous puparia products generally showed limited direct inhibition. These surviving counts support reduced growth under the tested conditions. Direct-assay solvent preparation and pH were not recorded and cannot be borrowed from the cell assay in the same study [20]. The adult BSF samples in this study were classified as non-by-products. Bleached homogeneous adult chitosan gave 2 × 10^10^ CFU/mL against *E. faecalis*, while the other adult treatment-organism combinations generally showed limited effects. These adult data provide comparative evidence for future residue processing, rather than results for dead-adult by-products [20].

For *Tenebrio molitor* exuviae, M1 used acid demineralisation, alkaline deproteinisation and deacetylation at 80 °C; M2 omitted demineralisation; M3 used pressurised deacetylation at 121 °C for 30 min. DDA/ M_v_ values were 42.62 ± 0.38%/71 ± 0.05 kDa, 25.25 ± 1.2%/97 ± 1.17 kDa and 55.62 ± 0.79%/116 ± 2.53 kDa, respectively. Prepared solutions contained 1% material in 0.5% acetic acid at pH 4.8; 5 mL was added to bacterial cultures, but the final concentration was unavailable. Viable counts at 35 °C after 24 and 48 h were assessed for *E. coli* ATCC 25922, *S. aureus* ATCC 25923, *E. aerogenes* ATCC 13048, *P. aeruginosa* ATCC 77853 and *Salmonella* spp. Inhibition generally increased with time, while *S. aureus* was relatively poorly inhibited, particularly by M2 at 24 h. Exact inhibition percentages and the bioassay comparator were not recorded, preventing quantitative ranking of extraction routes or a dose–response analysis [28].

Non-by-product evidence provides guidance for testing the methods described in Section 3, rather than measured bioactivity of residual streams. In adult *T. molitor*, chemical extraction and 60% NaOH deacetylation at 120 °C were followed by microwave depolymerisation at 800 W for 6, 18 or 30 min. The native material, the freeze-dried control (T0), T6, T18 and T30 had MW values of 492.49, 419.02, 378.17, 311.26 and 328.90 kDa, respectively, with DDA approximately 50–52%. Antimicrobial stocks contained 5 mg/mL in 0.5% acetic acid at pH 5.9, followed by serial dilution; commercial chitosans of different MW were comparators. Native/T18 MIC-MBC pairs were 0.16/0.16 versus 0.04/0.08 mg/mL for *Listeria monocytogenes*, 1.25/1.25 versus 0.63/0.63 for *S. aureus*, 2.50/5.00 for both against *E. coli*, and 0.08/0.63 versus 0.08/0.16 for *Salmonella* spp. T30 reproduced the T18 endpoints. However, T0 already showed lower *Listeria* MIC and *Salmonella* MBC than native material, so all changes cannot be assigned to irradiation. Strain identifiers, incubation duration, solvent-only outcomes and numerical commercial-comparator results were unavailable. This study supports testing organism-specific changes after processing by-product chitosan, not predicting uniform improvement from lower MW [34].

A second non-by-product adult-mealworm study compared solvent decolourisation with NaClO-based pretreatment, followed by 50% NaOH deacetylation at 100 °C for 3 h. DDA values were 75.84% and 72.6%; MW was unavailable. Autoclaved solutions of 4% and 8% chitosan in 1% acetic acid were applied at 20 µL/disc against *P. aeruginosa*, with ampicillin, commercial chitosan and PBS as comparators. The combined result was a reported 4 mm inhibition zone at 8% and no effect at 4%, without method-specific outcomes. This two-concentration observation is not an MIC, and the unspecified zone convention, assay pH, exposure time and matched acid-control result limit comparison with other studies [26].

Mechanistically, pH-dependent protonation of amino groups is a plausible basis for electrostatic interactions with microbial surfaces, but the assays above do not establish a common membrane-disruption pathway. Proposed nutrient-barrier effects of higher-MW material likewise remain interpretations unless directly tested. Neither inhibitory endpoints nor nanoparticle results demonstrate prevention of antimicrobial resistance, selective preservation of beneficial microbiota or replacement of antibiotics. The available comparisons also contradict a universal rule that increasing DDA necessarily increases antimicrobial activity [18,19,20,23,24,26,28,34].

### 5.2. Immunomodulatory Activity and Macrophage Engagement

The direct evidence concerns epithelial cells, not macrophages. In the homogeneous/heterogeneous BSF study described above, chitosan was dissolved in 17 mM acetic acid, adjusted to approximately pH 7 and diluted in DMEM. HaCaT keratinocytes received 0.5 mg/mL chitosan with or without 20 µg/mL *Salmonella* Typhimurium LPS for 6 or 24 h at 37 °C; HBD-2 gene expression was measured by qPCR, and commercial chitosan was the comparator. Unbleached homogeneous puparia chitosan produced the greatest 6 h induction among homogeneous samples, with sustained induction at 24 h. Bleached heterogeneous puparia material was the strongest heterogeneous sample and comparable to commercial chitosan. Numerical fold changes were unavailable, so the relative responses cannot be converted into effect sizes [20].

Adult BSF material classified as non-by-product showed greater HBD-2 induction for unbleached than bleached homogeneous preparations; heterogeneous adult effects were more limited. These results can guide testing of the corresponding Section 3 procedures on residual biomass, but do not establish equivalent adult-residue responses. HBD-2 transcription supports an epithelial innate-response hypothesis; it does not demonstrate increased secreted peptide, peptide-mediated bacterial killing, macrophage polarisation or systemic immunoprotection. TLR, Dectin-1, NF-κB and Th1/Th2 pathways were not demonstrated in the recorded experiments and are not assigned to the insect materials [20].

### 5.3. Haemostatic Capacity

Direct evidence extends beyond in vitro assays. BSF pupal shells were defatted, extracted with choline chloride-lactic acid DES at 92 °C for 5.6 h, bleached and partially deacetylated. The author-labelled powder series Cts1-Cts3 had DDA values of 36.3%, 41.85% and 65.3%, respectively; molecular masses were reported only as being of the order of 10^5^ g/mol. These materials are retained as a partially deacetylated series, with untreated chitin Cts0 identified solely as a comparator. Individual deacetylation conditions were unavailable. In the clotting assay, 6 mg powder was mixed with 100 µL blood and 10 µL 0.2 M CaCl_2_ for 60 s at 37 °C. Cts2 gave the lowest blood-clotting index, with the order Cts0 > Cts3 > Cts1 > Cts2; exact index values were unavailable [33].

In the rat femoral-artery bleeding model, 50 mg was applied topically, with three animals per group. Cts2 produced blood loss of 285.33 ± 37.05 mg, compared with 462.13 ± 42.56 mg for Celox and 534.53 ± 55.96 mg for Cts3. Haemostasis occurred at 2.17 ± 0.24 min with Cts2 versus approximately 3 min with Celox/Cts3. These are direct preclinical results for a defined insect-derived powder, not clinical veterinary evidence or a dose–response study. Enhanced platelet adhesion and fibrinogen adsorption were observed, while prothrombin time and activated partial thromboplastin time were largely unchanged. This combination supports local blood-component interactions but does not establish a universal coagulation-cascade mechanism. Cts2 outperforming the more deacetylated Cts3 also shows that DDA alone did not predict haemostasis [33].

### 5.4. Antioxidant Activity

Direct dose–response evidence is available for *T. molitor* larval-exuviae chitosan. After 2 M HCl demineralisation, 5% NaOH deproteinisation and 50% NaOH deacetylation at 105 °C for 5 h, the material had 72.27 ± 1.03% DDA and M_v_ of 612 kDa. In 1% acetic acid, concentrations of 2, 5 and 10 mg/mL gave DPPH scavenging of 11.96 ± 0.49%, 32.51 ± 1.39% and 62.75 ± 2.91%, respectively, after 30 min in the dark at room temperature. The reported IC_50_ was 7.92 mg/mL. A commercial-chitosan comparator was included, but its numerical response was unavailable. The concentration series demonstrates increasing scavenging within this cell-free assay; it does not demonstrate protection from oxidative injury in cells or animals [21].

For the homogeneously deacetylated BSF materials characterised in Section 5.1, the reported DPPH IC_50_ values were 12.1 ± 1.0 mg/mL for unbleached puparia chitosan and 7.9 ± 0.3 mg/mL after bleaching. Dead-adult values were 17.8 ± 0.2 and 7.5 ± 0.5 mg/mL, respectively. Samples were mixed with 6 × 10^−5^ M DPPH and incubated for 30 min in the dark at room temperature; solvent and commercial-chitosan controls were listed. However, every reported IC_50_ exceeds the recorded test range of 0.156–5 mg/mL. The values therefore require clarification of the concentration basis or extrapolation procedure before they can be treated as experimentally bracketed IC_50_ estimates. The lower reported values after bleaching suggest an association with processing, but do not isolate effects of DDA, MW or pigment removal [19].

The non-by-product adult-mealworm depolymerisation study in Section 5.1 tested 0.25–5 mg/mL chitosan in 0.5% acetic acid. DPPH IC_50_ values were 0.30 ± 0.07 mg/mL for native chitosan, 0.71 ± 0.04 for T0, 0.52 ± 0.02 for T6, 0.79 ± 0.03 for T18 and 0.71 ± 0.04 for T30. Commercial chitosans of different MW were comparators, but their numerical IC_50_ values and the assay incubation conditions were unavailable. Microwave treatment did not improve scavenging relative to native material, and the response was not monotonic with irradiation time. Together with the antimicrobial endpoints, this indicates that lowering MW may affect different bioactivities differently. It provides a cautious hypothesis for testing the Section 3 depolymerisation method on by-product chitosan, rather than a numerical prediction of its antioxidant activity [34].

The non-by-product BSF prepupal diet/bleaching and pupal microwave-extraction studies discussed in Section 3 contain no bioactivity assays [25,32]. Their methods can guide future residue processing, but characterisation alone cannot predict the direction of a bioactivity change. Direct antioxidant evidence remains preparation-specific and cell-free; tissue efficacy and mechanisms are unresolved, and commercial comparators do not establish insect material efficacy [19,21,34].

### 5.5. Biocompatibility and Enzymatic Biodegradation

Biocompatibility findings are model- and exposure-specific. The BSF study recorded 97–100% HaCaT viability after 24 h of chitosan exposure at the tested cell-assay concentration of 0.5 mg/mL. This supports short-term cytocompatibility in that keratinocyte system, not uniform safety across doses, formulations or administration routes [20]. For the DES-derived Cts series, haemolysis was assessed at 6 mg/mL for 3 h at 37 °C using Triton X-100 and saline controls; Cts2 gave less than 5% haemolysis. This is a blood-compatibility endpoint for that powder and does not substitute for local-tissue, systemic or repeated-exposure testing [33].

The same pupal-shell study provides direct degradation-related evidence: Cts2 was incubated at 15 mg/mL in PBS containing 10 µg/mL lysozyme at 37 °C, and approximately 40% of its mass remained after 12 days. Lysozyme is a glycosidic-bond-hydrolysing enzyme, not a protease; here the measured endpoint was remaining material mass under lysozyme exposure. An enzyme-free degradation result was not established in this comparison, nor were released-product identification or an in vivo resorption profile. Thus, the observation should not be converted into a physiological clearance rate or attributed entirely to enzymatic chain cleavage. It also does not establish a universal relationship between DDA, MW and degradation [33].

Mealworm-exuviae films provided a different endpoint. The M1 preparation showed weight losses of 2.46 ± 0.43% for films cast from 1.5% solution and 4.88 ± 0.41% for those cast from 1% solution. Without a recorded exposure medium, duration or enzyme condition, these losses cannot be interpreted as lysozyme-mediated biodegradation; matched weight-loss results for M2/M3 were unavailable [28]. These observations do not establish that degradation determines tissue-replacement time or that every insect-derived preparation is non-toxic.

Overall, the available evidence indicates that the bioactivity of insect-derived chitosan is influenced not only by species and biomass origin, but also by extraction conditions, physicochemical characteristics, formulation and assay design. To facilitate comparison across studies, Table 2 summarises the reported bioactivity of chitosan and nanochitosan derived from *Hermetia illucens* and *Tenebrio molitor*, including extraction conditions, material characteristics, experimental conditions and main outcomes. Studies using non-by-product biomass are also included for comparative purposes and are explicitly identified.

## 6. Uses of Chitosan in Veterinary Medicine

Chitosan is being investigated as a multifunctional veterinary biomaterial because it combines biodegradability, biocompatibility, antimicrobial activity, haemostatic effects, and immunomodulatory activity with broad processability. However, the veterinary relevance of chitosan depends on its formulation, material specifications and the setting in which it is tested.

Chitosan can be manufactured as hydrogels, films, membranes, sponges, fibres, nanofibres, nanoparticles, microparticles, granules, and porous scaffolds. These formats have been studied as wound dressings, tissue-engineering matrices, surgical materials, feed supplements, and carriers for drugs or biological molecules; the literature generally describes these uses as promising applications rather than uniformly established clinical technologies [68,69]. The animal studies discussed below used crustacean-derived chitosan or products whose biological origin was not reported.

### 6.1. Wound-Management Biomaterials

Experimental wound studies provide evidence for particular dressings rather than a uniform effect of chitosan. In rats, a medical-grade, crustacean-derived chitosan membrane with a degree of acetylation of 10% reduced wound area at day 3 to 60 ± 6% of its original size, compared with 78 ± 19% for Aquacel; the membrane degraded between days 7 and 14 [70]. In 12 horses with 24 experimentally created distal-limb wounds, a chitosan-impregnated dressing achieved 93.8% closure at day 42 versus 80.2% with gauze, and 93.6% granulation coverage at day 28 versus 72.4% [71]. No adverse effects were observed in that experiment, but biological origin and MW were unspecified; the dressing contained 2% (*w*/*w*) medical-grade chitosan with reported DDA of 85–90%. Conversely, a cotton-type chitosan dressing in hairless dogs produced no healing advantage and was associated with severe inflammation and impaired re-epithelialisation and granulation [72]. These contrasting findings preclude generalising tolerability across formulations or species.

Haemostatic dressings have also been tested in an experimental porcine femoral-artery injury model. Among ten pigs, chitosan fibre and sponge dressings achieved haemostasis in 3.25 ± 1.26 and 2.67 ± 0.58 min, respectively, with survival to 180 min in both treatment groups, compared with no survivors in the gauze group [73]. The groups were small (four, three and three animals), and the recorded biological origin, DDA and MW were unavailable. This study supports haemostatic performance under the tested injury and resuscitation conditions; it does not establish veterinary clinical effectiveness or safety for insect-derived chitosan.

Combination products require a separate assessment. In 24 dogs with experimental full-thickness wounds, platelet-rich plasma (PRP), PRP/chitosan nanoparticles and PRP/chitosan-silver nanoparticles were compared with saline over 21 days [74]. Treatments comprised 1 mL PRP alone or 1 mL PRP plus 1 mL nanoparticle preparation, injected once around the wound margins. At day 21, epidermal regeneration scores (mean ± SE) were 0.00 ± 0.00, 7.00 ± 0.00, 6.67 ± 0.58 and 7.00 ± 0.00 for saline, PRP, PRP/chitosan and PRP/chitosan-silver, respectively. Although the silver-containing formulation showed favourable bacterial-count and later wound-contraction results, these scores do not demonstrate an additional epidermal benefit from chitosan over PRP alone. Chitosan origin, DDA, MW and administered polymer mass were not reported, and inconsistent wound-area units prevent a reliable quantitative comparison of that endpoint.

Clinical evidence exists for products of unspecified or crustacean origin. In 41 working donkeys and horses with open hoof wounds, topical commercial-chitosan powder, 1 g on days 1, 3 and 7 and subsequently weekly, was compared with conventional povidone-iodine care [75]. At week 3, lameness scores of 0–1 occurred in 18/21 treated animals versus 4/20 controls. Horn maturation occurred at 11–13 weeks in 20 treated animals, with one severe case requiring 32 weeks, compared with 18–23 weeks in controls. These results concern the complete wound-care regimens, which differed in dressing and application schedules; the chitosan source and DDA/MW were unspecified, and dedicated adverse-effect assessment was not reported. A separate uncontrolled case involving one dog treated with shrimp-shell chitosan described clotting, fluid absorption and healing over four weeks [76]. Its design cannot establish treatment efficacy.

A preliminary clinical series also used gentamicin-loaded, cross-linked chitosan membranes for shell injuries in 12 Hermann’s tortoises [77]. All were reported to recover during the 21-day treatment period, although two developed mould requiring membrane removal and replacement. With no concurrent control, this observation cannot distinguish contributions from chitosan, gentamicin or associated care. None of these clinical reports provides evidence for insect-derived chitosan.

### 6.2. Scaffolds for Tissue Regeneration and Surgical Repair

Experimental repair studies show that improved histology does not necessarily translate into a measurable functional benefit. In nine dogs with paired buccal wounds, topical shrimp-derived chitosan lactate powder, reported DDA 92%, was compared with gauze [78]. Median bleeding times were 138 versus 309 s, but the difference was not statistically significant (*p* = 0.139); wound-size reduction also did not differ significantly (*p* = 0.210). Histological differences favoured treatment for selected measures of inflammation, collagenisation, granulation and re-epithelialisation over days 5–15. The fixed right-sided treatment and left-sided control allocation and small sample warrant caution when interpreting these findings.

Scaffold-based treatment was not consistently superior to simpler comparators. In immature teeth with apical periodontitis in four dogs, hyaluronic-acid/chitosan and pectin/chitosan scaffolds were compared with a blood clot and mineral trioxide aggregate apexification [79]. At 13 weeks, lesion resolution was 47%, 58%, 79% and 94%, respectively. Complete filling with vital tissue occurred in 0/19 hyaluronic-acid/chitosan-treated roots and 3/19 pectin/chitosan-treated roots, compared with 10/19 blood-clot-treated roots. The newly formed tissues included cementum-, bone- and connective-tissue-like structures rather than demonstrating restoration of normal pulp. These results provide no basis for assuming that adding a chitosan composite improves endodontic regeneration.

Other studies are less quantitatively conclusive. In 18 rabbits undergoing skin autografting, chitosan powder placed beneath the graft was associated with qualitatively thinner scars and more follicles, but all animals healed and a numerical between-group healing benefit was not reported [80]. Chitosan/bioactive-glass scaffolds were reported to achieve closure of diabetic rat wounds by day 14, but the extracted study record identified only one animal per wound-treatment group and inconsistent strain reporting [81]. Separate implantation experiments showed an early inflammatory response, illustrating that local responses depend on the composite and observation period. Collectively, these studies support further formulation-specific testing rather than clinical equivalence across chitosan scaffolds. No veterinary clinical or field study of tissue regeneration using insect-derived chitosan was identified.

### 6.3. Drug, Vaccine and Biological Delivery Systems

The reviewed delivery evidence comprises laboratory formulation studies, experimental animal assessments and secondary literature on vaccination. It does not establish clinical performance of an insect-derived carrier. In a veterinary skin-infection formulation study, commercial chitosan with DDA 85% and reported MW 235 kDa was prepared as 3% gels containing chlorhexidine and miconazole, with or without Tris/EDTA or chlorhexidine-loaded nanoparticles [82]. The experiments were in vitro, including bacterial and fungal assays, release measurements and L929-cell testing. At 6 h, the gel containing non-encapsulated drugs released approximately 98% of chlorhexidine and 22.6% of miconazole, whereas the nanoparticle-containing gel released 55.73 ± 7.04% and 43.44 ± 4.49%, respectively. Thus, changing the formulation altered the release of both agents. Antimicrobial activity belongs to these drug-containing systems and cannot be assigned to chitosan alone; in vitro viability results do not establish safety on animal skin.

Injectable systems likewise require assessment of the complete formulation. Thermosensitive chitosan/β-glycerophosphate hydrogels, administered as single 50 µL hindpaw injections in rats, induced acute oedema, with lower responses at 5 h for higher-DDA formulations [83]. This is a formulation-specific inflammatory response, not evidence that increasing DDA universally improves biocompatibility. The gentamicin-loaded membranes discussed above provide another delivery example: approximately 70% of gentamicin was released within 24 h in vitro, followed by slower release extending to 56 days [77]. That release profile should not be interpreted as a demonstrated 56-day therapeutic effect in tortoises.

A review of chitosan nanoparticle mucosal vaccines describes applications in poultry and pigs [84]. However, a review-level account does not substitute for a comparison of primary-study antigen doses, administration schedules, material specifications and quantitative protection outcomes. It therefore supports the existence of a research area rather than a conclusion about clinical effectiveness, reduced antigen requirements or lower treatment costs. No reviewed veterinary clinical or field study used a documented insect-derived chitosan delivery system.

### 6.4. Feed Additives and Dietary Supplementation

The available feeding studies span pigs, poultry, rabbits, lambs and dairy cattle, but their materials, doses and endpoints are heterogeneous. Reviews describe changes in digestibility, ruminal fermentation, feed conversion, growth, egg traits, immune indices and gut microbiota across polymeric-chitosan feeding studies [85].

COS studies represent a separate evidence stream. Examples include 200–600 mg/kg COS in weaner pigs, a 150 mg/kg COS plus *Clostridium butyricum* formulation in pigeon squabs, and COS supplementation in sows and poultry; COS products may also contain carriers such as cyclodextrin, so the observed response is not equivalent to polymeric chitosan [85,86]. Chitosan nanoparticles and mineral- or probiotic-loaded composites likewise require formulation-specific controls. Feeding studies using chitosan of insect-derived origin were not identified with sufficient material specifications to support a species-specific recommendation.

In vitro rumen or artificial-rumen experiments are not feeding trials. Chitosan has been tested as a rumen-fermentation modulator, with reports of altered volatile fatty acids, increased propionate and reduced methane under these systems; these findings require separation from in vivo digestibility and performance outcomes [85,86]. Similarly, chitosan treatment of poultry litter is an environmental-management intervention, not dietary supplementation. One review reports reduced nitrogen loss and ammonia-producing microorganisms after litter treatment with 10% *w*/*w* chitosan, but that result cannot be combined with dietary methane or growth outcomes [85].

The feed literature therefore supports application-specific testing rather than a general veterinary recommendation. Future trials should identify chitosan versus COS, origin and starting material, DDA, molecular-weight average and distribution, dose units, duration, basal diet, comparator, animal species, health status and adverse effects. They should also distinguish feeding trials from rumen simulations, litter treatments and cargo-loaded formulations.

Table 3 summarises the main chitosan-based biomaterials investigated for veterinary applications, including material origin, formulation, study design, comparators and principal outcomes across in vitro studies, experimental animal models and veterinary clinical studies. Together, these data provide an overview of the current evidence supporting the use of chitosan-based materials in veterinary medicine.

### 6.5. Safety and Batch Specifications

Insect-derived chitosan should be treated as a defined biological-material product rather than as a chemically uniform polymer. The relevant safety question is not whether insect chitosan is inherently allergenic or non-allergenic, but whether the specified batch contains residual insect or feed proteins, process contaminants, microbial hazards and formulation-related impurities at levels compatible with the intended veterinary use [35]. Purified chitosan cannot be assumed to retain the allergenicity of the starting tissue [92], but purification also cannot be assumed to remove every allergen or contaminant without product-specific testing [35].

Residual proteins are a particular concern because insect proteins can sensitise individuals and may cross-react with crustacean or mite allergens. EFSA assessments of whole or powdered insects identify primary sensitisation to insect proteins and possible reactions in people allergic to crustaceans, mites or molluscs; they also note that allergens from the feed may be transferred to the insect product [93,94]. These findings concern food materials containing protein, not purified chitosan, and therefore do not demonstrate allergenicity of the polymer used in this review. They do establish why residual-protein testing and substrate traceability are required. In chitosan preparations, total nitrogen cannot be converted to protein with a generic factor unless nitrogen intrinsic to the polymer is corrected; non-protein nitrogen from chitin can overestimate protein content [93].

The potential hazard profile also includes endotoxins, viable or non-viable microbial contamination, heavy metals, mycotoxins, pesticides, veterinary-drug residues, residual acids or alkalis, bleaching reagents, organic solvents and, for formulated products, cross-linkers, catalysts, stabilisers and loaded active ingredients. These hazards should not be presented as demonstrated contamination in the insect-derived chitosan studies reviewed here. The available insect-chitosan reports primarily measured yield, DDA, molecular weight, ash, moisture, protein or selected structural properties; they did not establish a common dataset for endotoxin, mycotoxin, microbial, residual-solvent or heavy-metal contamination. Separate analyses of insect feed materials have detected or identified possible hazards. Heavy-metal transfer depends on species, life stage, substrate and rearing conditions, while mycotoxins often show limited accumulation, but the evidence remains incomplete [95,96]. In BSF production samples, ICP-MS measurements found non-essential elements below the applicable limits in the tested materials, but this result concerns those sampled feed-chain matrices and cannot be transferred automatically to every chitosan batch [97].

Substrate composition must therefore be recorded from rearing through conversion. EFSA identifies substrate, species, developmental stage, harvest and further processing as determinants of contaminant occurrence and states that data on transfer from different substrates remain limited [98]. A waste-derived diet does not prove contamination of the final polymer, but neither does a purified spectrum prove its absence. Each batch should have traceable records for species, developmental stage, rearing substrate, collection point, storage conditions, extraction lot, reagents, washing and drying, deacetylation, sterilisation and packaging.

Application-specific release specifications should include identity and polymorph, DDA or DA with a validated method, molecular-weight average and distribution, residual protein, ash, moisture, elemental contaminants, endotoxin where parenteral or implantable use is contemplated, microbial limits, residual reagents and solvents, particle or pore attributes where relevant, and stability through the proposed shelf life [35]. The reviewed evidence does not establish a universal contaminant limit or a purity value that by itself proves biomedical suitability.

Sterilisation is part of the product specification, not a final administrative step. Heat and saturated steam can reduce aqueous solubility and hydrophilicity in chitosan acetate membranes, while gamma irradiation can cause main-chain scission and dose-dependent viscosity loss; heat exposure can also reduce solubility, increase colour and promote cross-linking [99]. In a PVA/chitosan antiseptic dressing, sterilisation altered degradation and was associated with densification, reduced porosity and increased cross-linking in one formulation, while effects differed with composition [100]. The selected sterilisation process should therefore be validated on the final formulation, with pre- and post-sterilisation comparison and stability testing. A material that is safe before sterilisation is not automatically safe or equivalent after sterilisation.

### 6.6. Manufacturing and Regulatory Translation

Manufacturing translation requires reproducible control of the starting stream and the conversion process. The principal risks are variable composition, intermittent by-product supply, inadequate drying or storage, heterogeneous particle size, incomplete deproteinisation or demineralisation, batch-to-batch DDA and molecular-weight variation, residual colour bodies, incomplete washing, reagent carry-over, microbial recontamination and inconsistent formulation. These risks are particularly relevant when dead adults or rejected insects are classified as by-products without documented provenance. A validated manufacturing process should define incoming-material controls, in-process controls, mass balance, critical process parameters, critical quality attributes, deviation handling, cleaning, segregation and traceability. GMP principles are relevant when the product is manufactured as a medicinal product or vaccine carrier; EU GMP guidance for human and veterinary medicinal products addresses contamination, cross-contamination and process variability [101,102].

The regulatory pathway depends on the product’s intended claim and jurisdiction. In the EU, a wound dressing for veterinary use requires classification according to its intended purpose and principal mode of action; it cannot be assumed to follow the same route as a veterinary medicinal product, and a chitosan carrier containing an active drug, antigen or vaccine is not regulated solely as an inert polymer. A veterinary medicinal product or immunological veterinary medicinal product requires product-specific quality, safety and efficacy assessment, pharmacovigilance and applicable manufacturing controls under Regulation (EU) 2019/6. A device classification depends on the intended purpose, mode of action and formulation. A feed use is subject to feed legislation and, where chitosan is presented as a feed additive rather than an ordinary feed material, the EU authorisation framework under Regulation (EC) No 1831/2003; authorisation should not be inferred from experimental feeding results [103]. EU insect-feed controls additionally address hygiene, contaminants, traceability, animal-by-product status and analytical identification of insect-derived proteins [104,105].

The EU rules for insects used in feed have developed through specific authorisations and restrictions rather than through a single pathway for all insect products. Insect proteins are treated within the processed-animal-protein framework, and authorised species, substrates, animal categories and analytical controls depend on the applicable legislation [106,107]. The reported ability to detect prohibited ruminant material in BSF feed chains using complementary molecular and mass-spectrometric methods illustrates the importance of substrate and species traceability, but it does not constitute a regulatory approval for chitosan manufacture [108].

For veterinary translation, development should proceed by product class: (i) topical wound dressing, with material identity, sterility, local tolerance, degradation and classification assessed for the intended claim; (ii) medicinal product, with GMP, quality, safety, efficacy and pharmacovigilance requirements; (iii) drug or vaccine carrier, with characterisation of both polymer and cargo, release, potency, sterility and immunotoxicity; and (iv) feed or feed additive, with substrate traceability, contaminant and microbiological control, species-specific safety and the relevant EU authorisation route. Human-device, food-novelty and feed rules cannot be assumed to govern veterinary products identically. The principal requirements are binding only when the product falls within the relevant legal category; GMP, validated analytical methods, stability studies and formulation-specific risk assessment are development requirements whose exact scope depends on the route and claim [101,103,104].

The reviewed evidence supports a development framework, not a claim of regulatory approval. A reproducible insect-derived chitosan product would require a documented chain of custody, validated analytical methods, defined batch specifications, demonstrated sterilisation compatibility, stability data, toxicological and local-tolerance studies, and manufacturing controls proportionate to the intended route. Only after those product-specific data are generated can an environmental by-product claim be connected credibly to a safe, consistent and legally marketable veterinary material.

## 7. Sustainability and Industrial Feasibility

The environmental case for insect-by-product chitosan depends on the complete chain from insect production to purified polymer. Insect rearing, by-product collection and storage, and chemical or biological conversion are separate sources of burden and should not be combined implicitly. Crustacean shells provide an important comparator because they are processing residuals from the marine food industry whose share of upstream environmental burdens depends on the allocation method; reported shell chitin content is commonly 15–40%, although composition and supply vary [2]. An insect side stream may avoid a new biomass harvest, but this advantage is conditional on its actual collection, transport, drying, storage and conversion requirements.

### 7.1. Burden Allocation and Material Supply

For *H. illucens* and *T. molitor*, chitin-rich residues arise alongside intended protein, lipid, frass and other products. The environmental burden of rearing should therefore be allocated among these outputs using an explicitly stated method, such as mass, economic or system-expansion allocation. The studies cited in this review do not provide a harmonised allocation analysis. Consequently, it is not possible to assign the full insect-rearing burden to chitosan, or to claim that chitosan from a residual stream is environmentally superior to chitosan from crustacean shells without a common functional unit and system boundary.

Material availability is also not equivalent to theoretical chitin content. Pupal exuviae and larval exoskeletons can be collected from established insect-processing operations, whereas dead adults, rejected insects and whole insects require documented provenance before they can be treated as by-products. *H. illucens* pupal exuviae used in one study were manually collected from a farm and stored at −18 °C; the insects had been reared on plant-origin food-processing by-products [7]. This demonstrates a traceable starting stream, but it does not quantify the energy or emissions associated with freezing, drying, milling, storage or transport. *T. molitor* breeding rejects, and adult processing residues have likewise been studied, but their availability, collection frequency, moisture at collection and storage stability were not reported in a form that supports an industrial supply estimate [4,16].

### 7.2. Chemical, Water and Energy Requirements

Conventional conversion can require sequential defatting, demineralisation, deproteinisation, decolourisation, washing, drying and alkaline deacetylation. In *H. illucens* larval exoskeletons, formic acid consumption was reported as 5 mol kg^−1^ starting exoskeleton, with 89% demineralisation effectiveness; heterogeneous deacetylation at 120 °C gave a 47% conversion yield relative to chitin applied in Table 3 of the source, whereas the homogeneous route at 4 °C gave 13% [15]. These values show process severity and conversion differences, but they are not an environmental comparison with crustacean processing.

For *H. illucens* puparia, an optimised chemical process reported 250 ± 26.86 L kg^−1^ water consumption, a total reaction time of 1.15 ± 0.08 h, 94.25 ± 0.6% deproteinisation efficiency, 34.74 ± 1.15% chitin yield and 83.33% chitosan yield relative to chitin [29]. The study provides useful process measurements, but the reported water value, yield denominators, product specifications and system boundary cannot be used to calculate a comparative environmental advantage over crustacean shells. It also does not provide a complete energy balance, effluent-treatment burden or cost.

Drying and washing may be consequential at scale. Insect residues can require drying before milling and repeated washing after acid, alkali or bleaching treatments; deacetylation commonly uses concentrated alkali at elevated temperature. These operations generate neutralisation streams containing salts, dissolved proteins, pigments and residual reagents. The reviewed primary studies generally report product yield and selected composition rather than the chemical oxygen demand, salt load, toxicity, treatment energy or disposal route of their effluents. A lower reagent concentration in one step therefore cannot be interpreted as a lower total impact for the process.

### 7.3. Biological and Emerging Solvent-Based Routes

Biological processing may reduce nominal temperature or avoid some mineral acids, but it can require enzyme manufacture, fermentation time, pH control, water, downstream separation and sterilisation. In *T. molitor* adult processing residues, enzyme-assisted deproteinisation achieved 86.1–94.1% efficiency, with residual lipid of 0.9–4.8 g/100 g, ash of 0–0.5 g/100 g and approximately 7% moisture; chemical deacetylation still followed and produced chitosan with 85% DDA [16]. The study does not provide a full energy, water, enzyme-production or effluent inventory, so the biological route cannot be ranked as lower impact on that basis.

Deep eutectic solvents and microwave-assisted methods provide measured process advantages in particular experiments. Microwave-assisted choline chloride-lactic acid extraction of skimmed BSF produced 3.8% chitin relative to starting material in 1 min, recovered 7.4% protein to the supernatant and recovered approximately 80% of the solvent for reuse [109]. These are relevant fractionation and solvent-recovery results, but solvent recovery is not the same as demonstrated life-cycle benefit; the study does not report repeated recovery cycles, solvent losses, electricity demand, equipment cost or a complete chitosan route. Similarly, DES extraction of *H. illucens* pupal shells produced 28.40% chitin at 96.93% purity under an optimised condition, but the available evidence does not establish its total water, energy, solvent or waste-treatment burden [33].

Other insect studies illustrate biorefinery potential without providing a direct comparison for the target residual streams. Ionic-liquid pretreatment of *Bombyx eri* larval chitin was coupled to enzymatic degradation and fermentation, and selective recovery of N-acetyl-D-glucosamine was reported at 899.2 mg g^−1^ of chitin for a two-step route versus 760 mg g^−1^ for the comparator [110]. This is evidence for integrated processing of another insect material, not proof that ionic liquids, fermentation, or a similar sequence lower the impact of *H. illucens* or *T. molitor* by-product chitosan. Solvent toxicity, recovery, residual solvent, enzyme demand and fermentation burdens require assessment.

### 7.4. Scale-Up, Quality and Cost

The largest directly stated scale in the primary evidence reviewed here is the 10 L deproteinisation scale used for *H. illucens* larval exoskeletons, where chitin content was 87% compared with 83% at small scale [15]. Other studies describe laboratory-scale extraction or optimisation experiments; the availability of a farm side stream does not demonstrate an operating chitosan plant. A review of insect extraction methods identified persistent gaps in purification degree, chitosan yield and quantitative analytical characterisation [111], while a sustainability assessment concluded that no reviewed workflow achieved a fully green conversion to chitosan and that sustainability data still need to be generated [1].

Scale-up bottlenecks include collection of clean and sufficiently dry residues, separation of developmental stages, storage stability, variable lipid and protein content, pigment load, particle-size control, reagent dosing, heat and mass transfer, washing-water recycling, solvent recovery, effluent treatment and batch release. These variables can alter DDA, molecular weight, crystallinity, residual protein, ash, moisture, colour and bioactivity. A material that meets one endpoint at laboratory scale is not necessarily a reproducible feedstock for a defined veterinary product.

Cost evidence is similarly incomplete. The reviewed studies report selected reagent, water, time, yield or solvent-recovery values, but not a harmonised techno-economic analysis covering feedstock collection, drying, labour, capital equipment, energy, waste treatment, quality assurance, depreciation and product purification. No reliable cost comparison between insect-by-product chitosan and crustacean-derived chitosan can therefore be stated. Reported yields are also incompatible when one study expresses chitin relative to starting biomass and another expresses chitosan relative to isolated chitin [2,29].

By-product valorisation could deliver environmental and economic advantages when the residual stream is genuinely generated by an existing insect operation; collection and storage require little additional material and energy; the stream is sufficiently concentrated and consistent; the extraction route recovers protein, lipid, frass and chitin products without double-counting benefits; reagents and water are recycled; effluents are treated; and the final chitosan meets a defined specification at reproducible yield. Demonstrating those conditions requires matched life-cycle and techno-economic studies with transparent allocation, functional units, system boundaries and product specifications. Until then, insect-farming by-products should be described as a conditional valorisation opportunity rather than as a guaranteed environmental or economic improvement.

## 8. Conclusions

Insect-farming by-products, particularly those generated by *Hermetia illucens* and *Tenebrio molitor*, represent a promising alternative source for chitosan production. Their valorisation can integrate chitosan recovery into existing protein- and lipid-production chains, reduce the amount of biological waste requiring disposal, and support circular-economy approaches [4,9,10]. Available evidence shows overlap in selected physicochemical measurements between insect-derived preparations and commercial crustacean-derived comparators; however, yield, purity, molecular weight, degree of deacetylation, morphology, and biological performance remain strongly dependent on the insect source and extraction conditions [3,23,24]. Insect-derived chitosan should therefore be treated as a product requiring defined specifications and batch-level characterisation, rather than as an automatically interchangeable material.

The functional value of chitosan, and, by extension, of insect-derived chitosan intended for veterinary use, is determined by the relationship between its chemical structure, physicochemical properties, and formulation. Degree of deacetylation, molecular weight and distribution, crystallinity, moisture, ash, residual protein, porosity, cross-linking, and sterilisation conditions can all influence performance and must be selected according to the intended application [35,57,67]. Chitosan’s antimicrobial, haemostatic, immunomodulatory, antioxidant, biodegradable, and processable properties support its development as a veterinary biomaterial, though the maturity of evidence varies considerably across applications. Veterinary wound-management, delivery, tissue-repair and feed studies provide formulation-specific evidence across laboratory, animal-model and selected clinical settings; these findings cannot be transferred automatically to insect-derived chitosan [68,92,112,113].

By bringing together the compositional and structural determinants of chitosan performance with the specific requirements of veterinary applications, this review identifies the evidence supporting further development and the limits to transferring findings between sources, formulations and species. Translation into reliable veterinary products will require reproducible extraction and manufacturing, validated analytical methods, product-specific assessment of residual proteins and other impurities, and systematic evaluation of toxicity, biocompatibility, degradation, mechanical performance, dose, release behaviour, and sterilisation. Studies should also use clinically relevant animal models and controlled trials that distinguish species, administration route, formulation, and intended outcome [3,92,113,114]. No veterinary clinical or field study using chitosan of documented insect origin was identified in the reviewed literature. Wound dressings and localised delivery systems are candidates for further investigation, but their clinical efficacy and environmental advantage remain to be demonstrated for defined insect-derived products. Its clinical adoption will ultimately depend on demonstrating consistent quality, safety, and efficacy for each defined product and use, and on confirming that its production remains genuinely advantageous within the broader circular-bioeconomy framework that motivates its use.

## Figures and Tables

**Figure 1:**
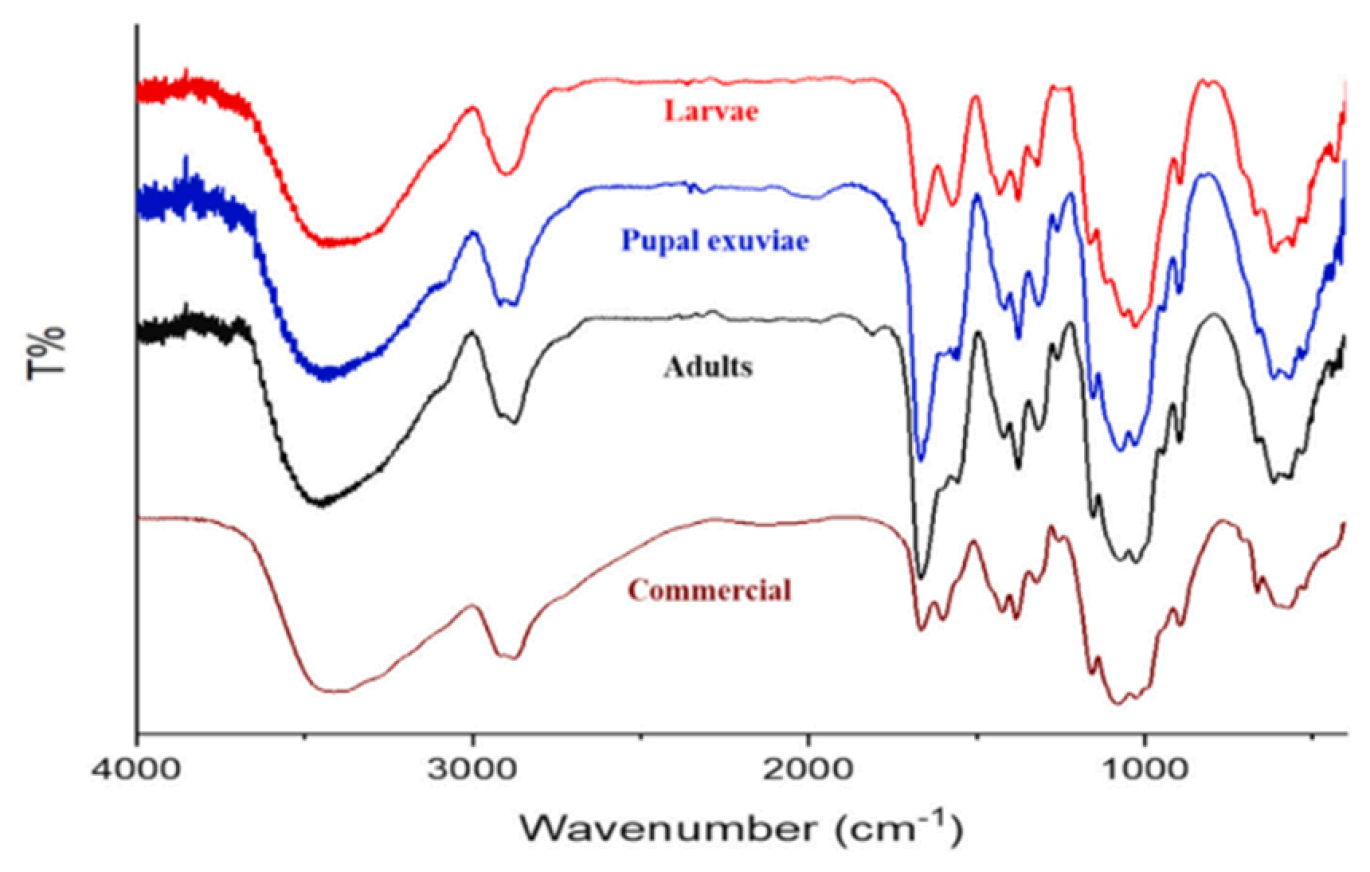
Representative FTIR spectra of chemically extracted chitosan from *Hermetia illucens* larvae, pupal exuviae (puparia) and adults, compared with commercial chitosan. Adapted from Triunfo et al. [19] under the CC BY 4.0 licence (https://creativecommons.org/licences/by/4.0/ accessed on 6 October 2026).

**Figure 2:**
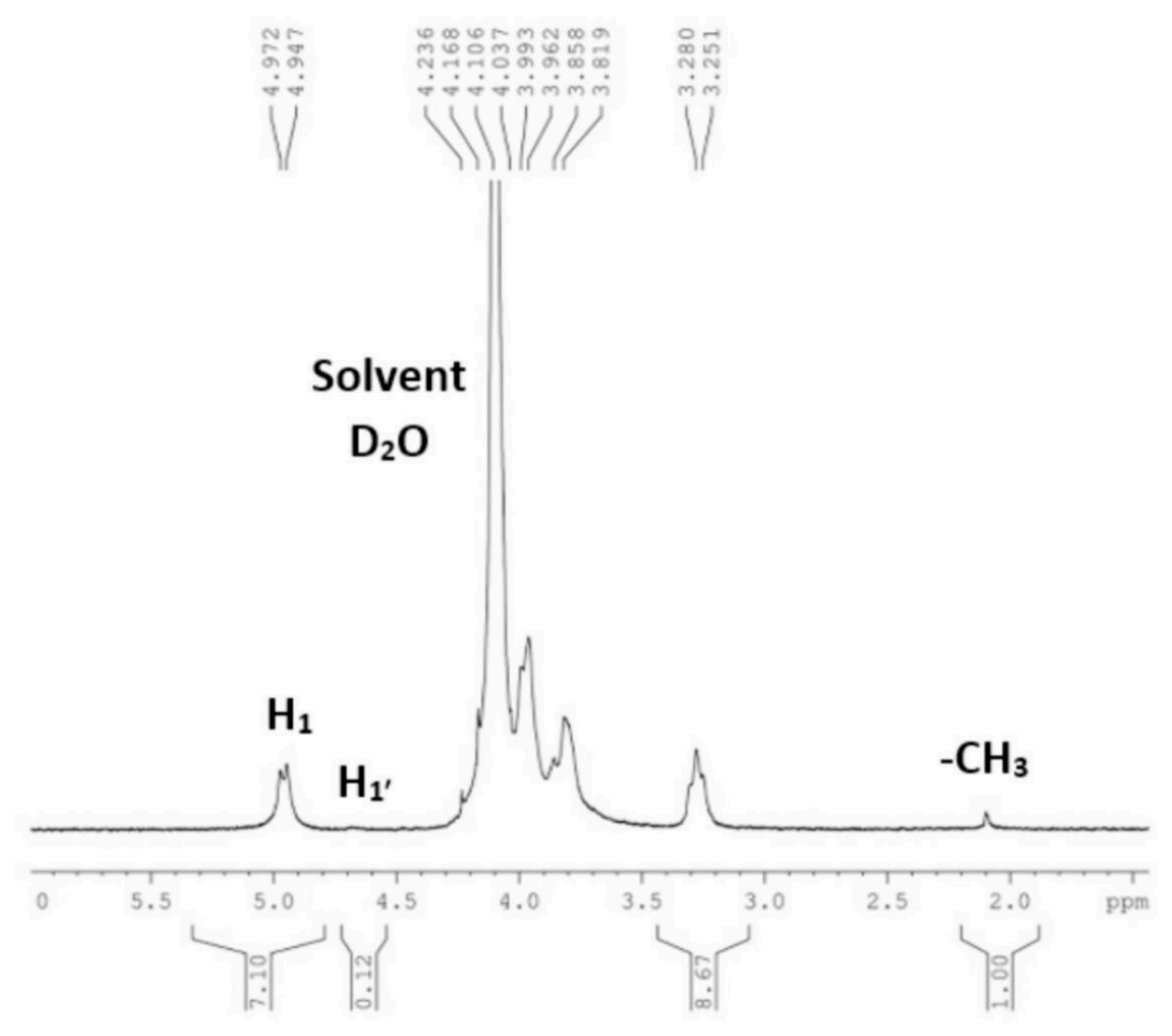
Representative ^1^H NMR spectrum of chemically extracted chitosan from dead adults of *Tenebrio molitor*. Adapted from Azzi et al. [4] under the CC BY 4.0 licence (https://creativecommons.org/licences/by/4.0/ accessed on 6 October 2026).

**Figure 3:**
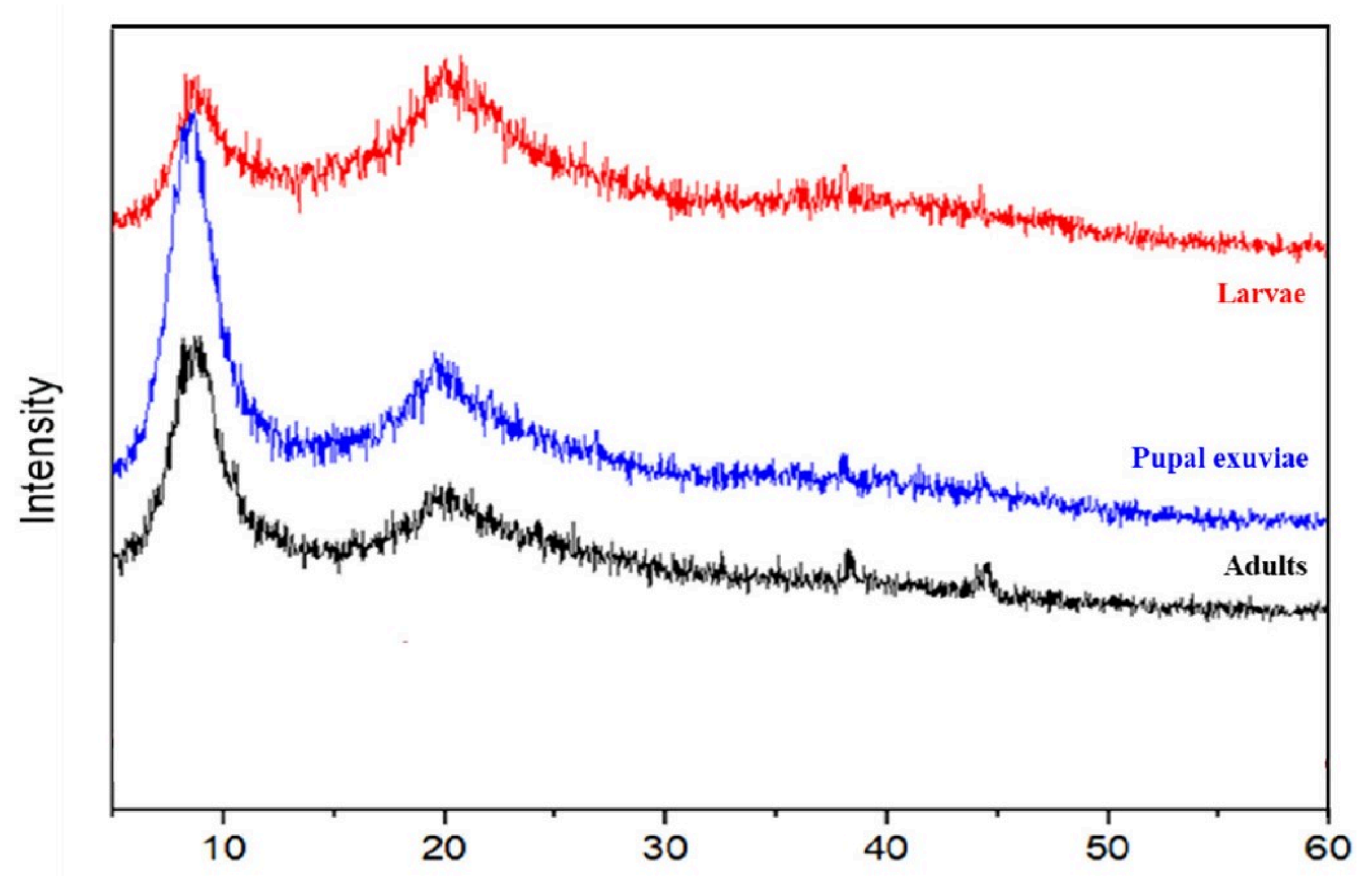
X-ray diffraction patterns of chemically extracted chitosan from *Hermetia illucens* larvae, pupal exuviae (puparia) and adults. Adapted from Triunfo et al. [19] under the CC BY 4.0 licence (https://creativecommons.org/licences/by/4.0/ accessed on 6 October 2026).

**Figure 4:**
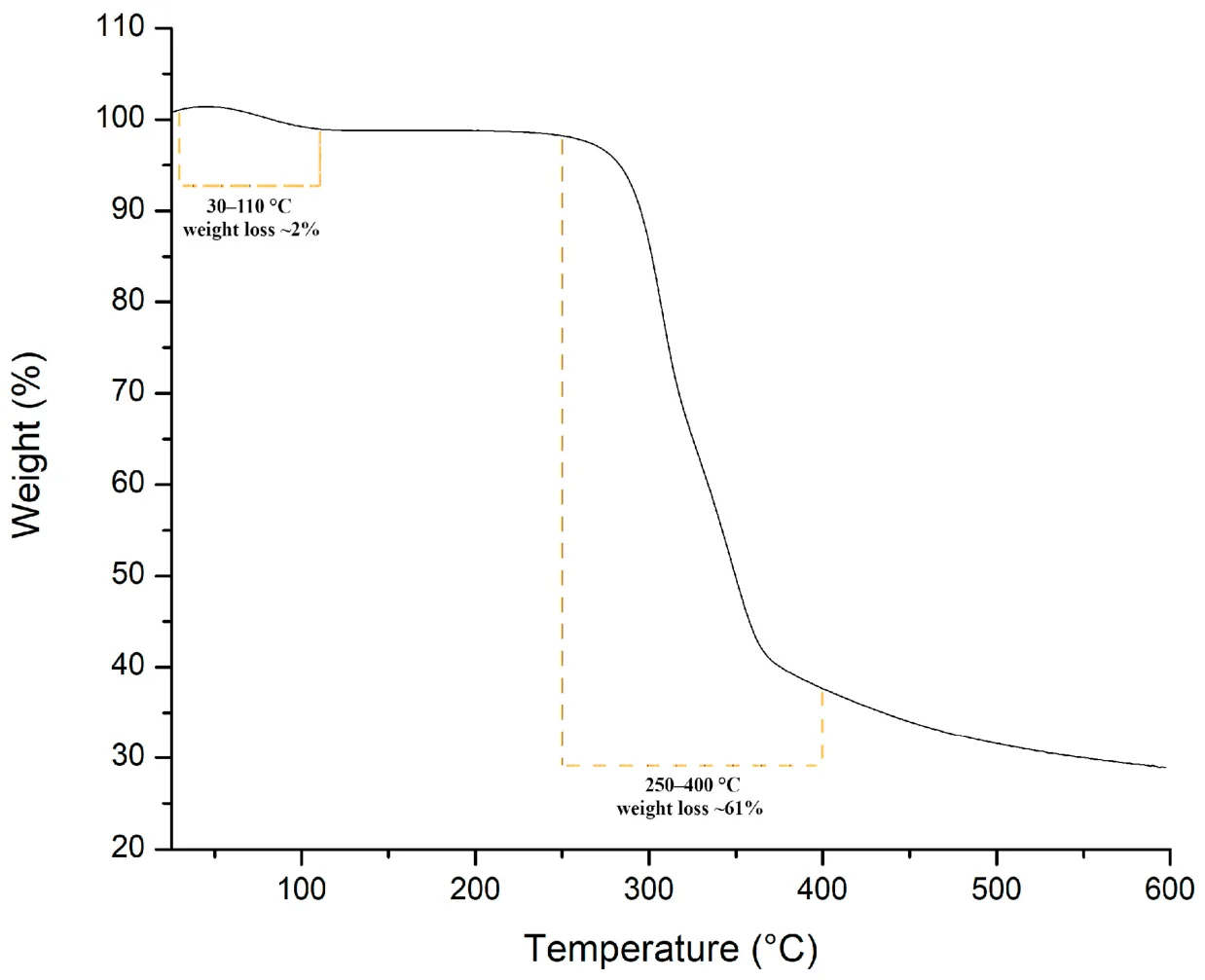
TGA profile of unpurified chitosan derived from *T. molitor* exuviae. Adapted from Budinčić et al. [21] under the CC BY 4.0 licence (https://creativecommons.org/licences/by/4.0/ accessed on 6 October 2026).

**Figure 5:**
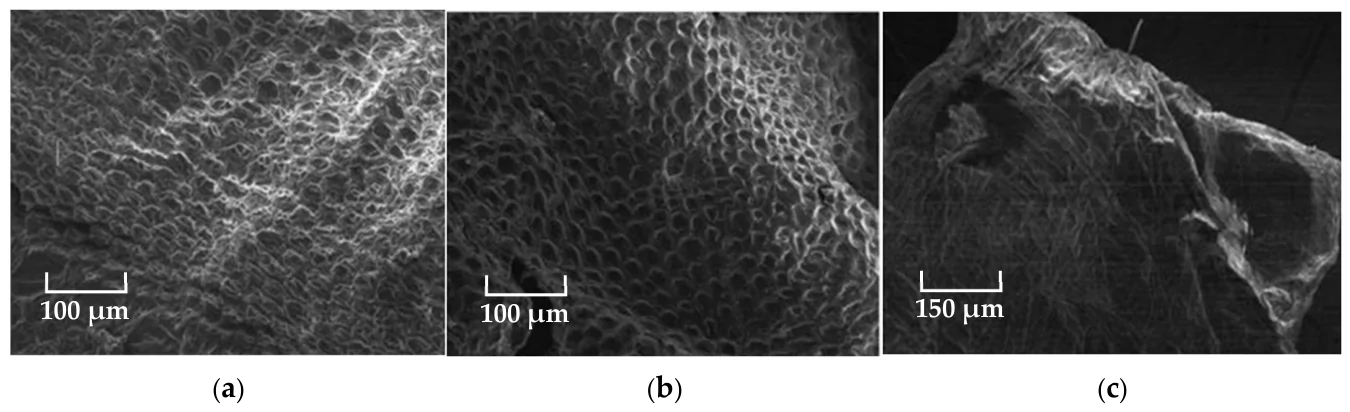
SEM images of chitosan derived from *H. illucens* exuviae ((**a**); magnification 500×), puparia ((**b**); magnification 500×) and adults ((**c**); magnification 450×). Adapted from Elkadaoui et al. [13] under the CC BY 4.0 licence (https://creativecommons.org/licences/by/4.0/ accessed on 6 October 2026).

**Figure 6:**
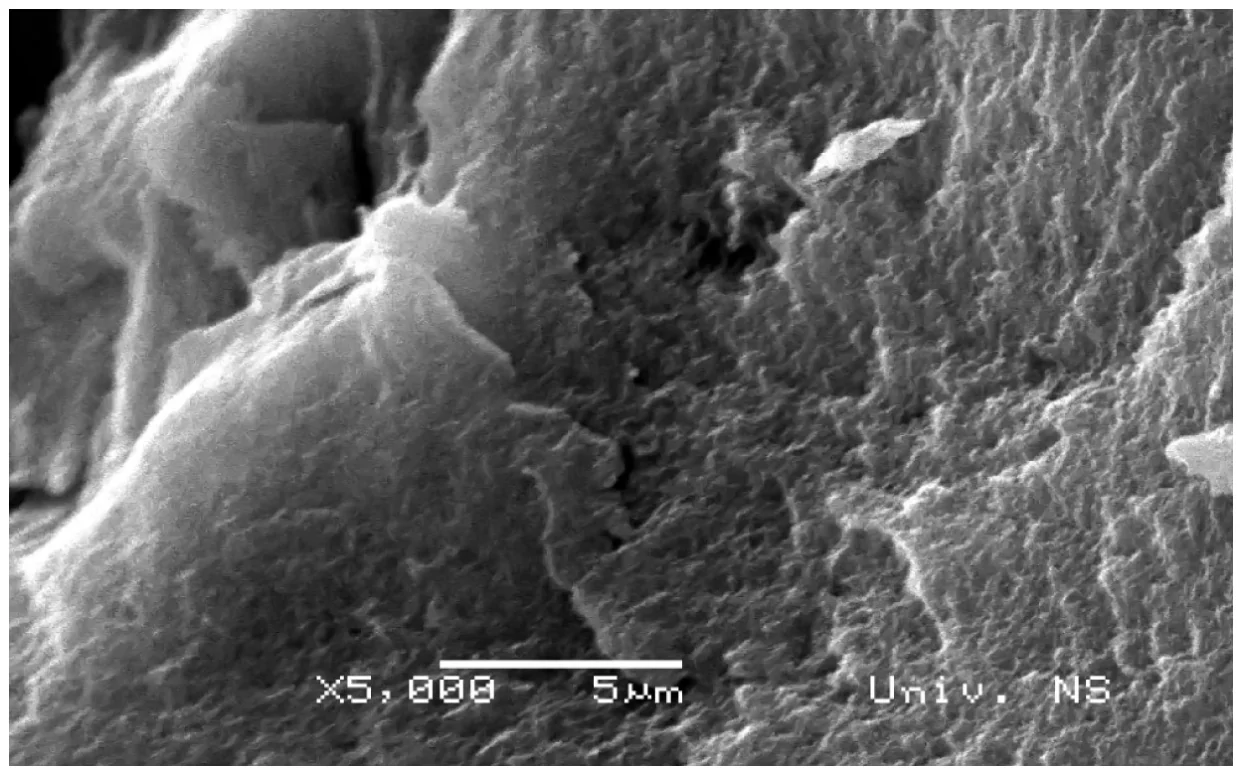
SEM images of purified chitosan derived from *T. molitor* exuviae (magnification 5000×). Adapted from Budinčić et al. [21] under the CC BY 4.0 licence (https://creativecommons.org/licences/by/4.0/ accessed on 6 October 2026).

**Table 1:** Extraction conditions, yields and physicochemical properties of chitosan derived from *Hermetia illucens* and *Tenebrio molitor* by-products. Studies using non-by-product biomass are included for comparison and explicitly identified.

Extraction Conditions	Rearing Substrate and Age	Chitosan Yield (%)	DDA (%)	MW (kDa)	Other Characterisation	Ref.
*Hermetia illucens*|Exuvia|Chemical extraction						
Dm: 0.5 M formic acid, RT, 1 h; Dp: 2 M NaOH, 80 °C, 2 h; Het Da: 12 M NaOH, N_2_, (Het1: 140 °C/3 h; Het2: 140 °C/6 h; Het3: 120 °C/3 h); Hom: 10 M NaOH, 4 °C/12 h; Het pur: 1% acetic acid, 80 °C/20 h, filtered and precipitated.	Larvae reared on agro-industrial feedstocks	Cs/raw: Het1: 8% Het2: 13%; Het3: 16%; Hom: 4% Cs/Ct: Het1: 22% Het2: 38% Het3: 47% Hom: 13%	Titration Het1: 44% Het2: 72%; Het3: 43%; Hom: 34% Het1, Het3 and Hom partially deacetylated materials reported as chitosan by the authors	NR	Dynamic viscosity: Het1: 31; Het2: 77; Het3: 277; Hom: 509 mPa·s (control 520). Film thickness: 28, 31, 29, 31 µm (control 28).	[15]
Dm: 0.55 M HCl, RT, 15 + 30 min; Dec: 33% H_2_O_2_/NaHCO_3_ (9:1, *w*/*w*), RT, 30 min; Dp: 1 M NaOH, 80 °C, 4–5 × 45 min; 1:10 (*w*/*v*). Da: 120 °C: KOH:ethanol:MEG 50:25:25, 24 h; pur: 0.5 M acetic acid, filtered and precipitated.	Larvae: rabbit feed (70% water), then fruit/vegetable waste LE1: 4–6 LE2: 8–12 d.	Cs/raw: 17–26.6% Cs/Ct: 71–73%	Titration 93–95% ^1^H NMR 98–100%	M_v_ 94–109 kDa pur: 95–110 kDa	Unpurified product: 3–10% insoluble impurities, removed by purification. SEM: Porous structure retained after deacetylation; purification removed visible impurities, giving well-organised pores.	[13]
*Hermetia illucens*|Exuvia|Emerging solvent and intensified processes						
Microwave-assisted 600 W: Dl: methanol:chloroform (1:4, *v*/*v*), 2 min; Dm: 0.55 M HCl (1:20, *w*/*v*), 4 min; Dp: 1 M NaOH (1:20), 6 min/bath × 5; Da: KOH:ethanol:MEG (50:25:25), 1:60 (*w*/*v*), 20 min	NR	Cs/raw: 21.14% Cs/Ct: 65.89%	^1^H NMR 97.7%	M_v_ 155 kDa	CrI: 51.26%; DTGmax 326 °C; Solubility in 0.3% acetic acid; intrinsic viscosity 687.2 mL/g. SEM: Deacetylation disrupted the ordered chitin structure	[30]
*Hermetia illucens*|Puparia|Chemical extraction						
Dm: 0.5 M formic acid (1:10, *w*/*v*), RT, 1 h; Dp: 2 M NaOH (1:10), 80 °C, 2 h; Dec: 5% (*v*/*v*) H_2_O_2_ (1:20–30), 90 °C, 30–60 min; Da: 12 M NaOH (1:20), 100 °C, 4 h; Pur: 1% (*v*/*v*) acetic acid, RT, 48 h, filtered and precipitated.	Gainesville diet	Cs/raw: Dec: 10 ± 0.2%; NoDec: 8 ± 0.8%. Cs/Ct: Dec: 42 ± 1.5% NoDec: 28 ± 4.5%,	Titration Dec: 90 ± 4.2%; NoDec: 83 ± 3.1%	M_v_ Dec: 35 ± 4.3 kDa; NoDec: 55 ± 1.7 kDa	CrI: Dec: 80%; NoDec: 78%; Films: both uniform; Dec lighter/clearer, unbleached transparent-translucent. SEM: Dec: less fibrillated, more homogeneous, porous; NoDec: rough, porous, more fibrillated.	[2]
Dm: 3 M HCl, 25 °C, 1 h; Dp: 1 M NaOH, 25 °C, 1 h (1:20, *w*/*v*); Dec: with 12% NaOCl, 10 min; Da: 50% (*w*/*v*) NaOH, 80 °C, 1 h	Rearing substrate: organic waste;	Cs/Ct: 36%	FTIR 53%	NR	CrI: 57%; Moisture 4.41%; ash 7.45%; Light-brown powder.	[22]
Dm: 0.55 M HCl, RT, 15 + 30 min; Dec: 33% H_2_O_2_/NaHCO_3_ (9:1, *w*/*w*), RT, 30 min; Dp: 1 M NaOH, 80 °C, 4–5 × 45 min; 1:10 (*w*/*v*). Da: 120 °C: KOH:ethanol:MEG 50:25:25, 24 h.	Larvae: rabbit feed (70% water), then fruit/vegetable waste	Cs/raw: 32% Cs/Ct: 74%	Titration 91% ^1^H NMR 96%	M_v_ 115 kDa.	Soluble in acetic acid; purification not required. SEM: Porous structure retained after deacetylation.	[13]
Dm: 0.5 M formic acid, RT, 1 h; Dp: 2 M NaOH, 80 °C, 2 h; Dec: 5% H_2_O_2_, 90 °C, 60 min; Da: 12 M NaOH, 4 °C overnight; ice, centrifugation.	NR	Cs/raw: No Dec: 3 ± 0.8%; Dec: 7 ± 2.4% Cs/Ct: No Dec: 10.9 ± 2.4%; Dec: 33 ± 7.8%;	Titration No Dec: 62 ± 3.5%; Dec: 72 ± 3.6%	M_v_ No Dec: 285 ± 29 kDa; Dec: 115 ± 16 kDa	CrI: NoDec: 51%; Dec: 68%; SEM: Dec: fewer surface particles and a more uniform surface than NoDec.	[19]
Dp 5% NaOH, (1/15, *w*/*v*), 90 °C, 4 h Dm 4% HCl (1/15, *w*/*v*), RT, 12 h Dec: 1% KMnO_4_; (1/20, *w*/*v*), RT, 12 h Da: 70% NaOH, 80 °C, 8 h	NR	Cs/raw: 27.64%	Titration 72.3%	M_v_ 194.9 kDa	Protein 0.29%; ash 0.03%; Viscosity 14.3 cP; Solubility 93.2%	[23]
Dm: 1 M HCl, 10 mL/g, RT, 1 h; Dp: 1 M NaOH, 25 mL/g, 80 °C, 13 × 1 h; Da: 50% NaOH (1:30, *w*/*v*), 90 °C, 10 h; Nanochitosan: 3% CS/2% acetic acid, 24 h; 250 mL STPP into 250 mL CS, 1000 rpm, 45 min; pH 5 (0.4 M NaOH/100 µM NaCl);	vegetable kitchen waste plus moist rice:wheat bran (1:1)	NR	FTIR bulk: 62.3% nano: 85.3%	NR	CrI: bulk: 77.2%; nano: 71.4%; DTGmax: bulk: 461.9 °C; nano: 462.3 °C residue at 600 °C: bulk: 14.7; nano: 17.7%; nano DLS ~300 nm zeta +42.3 ± 1.2 mV; whiteness: bulk: 56.7 ± 0.4; nano: 67.0 ± 0.4. SEM: Bulk: rough honeycomb/hexagonal surface with fibres and large pores. Nano: rough, dense particulate/lamellar aggregates;	[24]
*Hermetia illucens*|Puparia|Emerging solvent and intensified processes						
Dl: P1 none; P2 hexane/Soxhlet (1:20), 60 °C, 1 h; P3/P4 methanol:chloroform (1:4; 1:20), 25 °C, 1 h. Dm: 0.55 M HCl (1:10), 25 °C, 1 h. Dp: P1-P3 1 M NaOH, 80 °C, 18 h (1:20); P4 1 M NaOH (1:20), 121 °C/2.2 bar, 5 × 15 min. Da: KOH:ethanol:monoethylene glycol 50:25:25; P1-P3 120 °C, 24 h (1:60); P4 (AA): 121 °C/2.2 bar, 1 h (1:10).	Chick feed (Alf Sahel) with 70% water	Cs/Ct: P1: 68.57 ± 1.15%; P2: 72.02 ± 0.57%; P3: 74.37 ± 0.66%; P4: 83.33 ± 1.15%.	^1^H NMR P1 94.96 ± 0.02%; P2 95.23 ± 0.20%; P3 96.15 ± 0.14%; P4 97.08 ± 0.08%. FTIR P1 90.91 ± 0.18%; P2 93.67 ± 0.05%; P3 93.65 ± 0.78%; P4 94.20 ± 0.07%.	M_v_ P1 166.964 kDa; P2 95.316 kDa; P3 109.874 kDa; P4 220.378 kDa	CrI: P1 34.8%; P2 36.23%; P3 45.3%; P4 47.7%.; DTGmax: P1 323 °C; P2 338 °C; P3 343 °C; P4 345 °C.; SEM: All: relatively uniform overall structures, heterogeneous surfaces with disorganised microfibrils; lighter pores particularly evident in P2 and P3.	[29]
Dl: petroleum ether, Soxhlet, 55 °C, 6 h; dried at 110 °C; DES extraction: choline chloride-lactic acid, 92 °C, 5.6 h, 20 mL/g; Dec: 20% H_2_O_2_, pH 7.5, until pale yellow; Da: 1:30 NaOH: Cts1: 60-mesh, 33% NaOH, 90 °C, 3 h; Cts2: 80-mesh, 33% NaOH, 90 °C, 3 h; Cts3: 80-mesh, 42% NaOH, 120 °C, 4.5 h.	NR	NR	Titration: Cts1 36.3%; Cts2 41.85%; Cts3 65.3%. ^1^H NMR: Cts1 38.2%; Cts2 45.8%; Cts3 70.8%. Cts1 and Cts2: partially deacetylated materials reported as chitosan by the authors	M_v_ Cts1: 203.4 kDa; Cts2: 193.0 kDa; Cts3: 138.7 kDa.	Crystallinity: decreased with increasing DDA. Particle morphology: Cts1: >100 µm; Cts2: >100 µm, smaller than Cts1; Cts3: >100 µm. Zeta potential (3 mg/mL, deionised water): Cts1: positive; Cts2: positive; Cts3: highest positive potential;	[33]
*Hermetia illucens*|Dead adults|Chemical extraction						
Dm: 0.55 M HCl, RT, 15 + 30 min; Dec: 33% H_2_O_2_/NaHCO_3_ (9:1, *w*/*w*), RT, 30 min; Dp: 1 M NaOH, 80 °C, 4–5 × 45 min; 1:10 (*w*/*v*). Da: Broussignac (B): 120 °C: KOH:ethanol:MEG 50:25:25, 24 h; Kurita (K): 50% NaOH, 24 h	Larvae: rabbit feed (70% water), then fruit/vegetable waste.	Cs/raw: B24h: 9.5% K24h: 8.3% Cs/Ct: B24h: 69% K24h: 74%	^1^H NMR B: 4 h: 91%; 6 h: 93%; 8 h: 93%; 10 h: 94%; 24 h: 97%. K: 10 h: 92%; 12 h: 96%; 3 × 3 × 4 h: 100%; 24 h: 97%. Titration B: 24 h: 94%	M_v_ B: 4 h: 188 kDa; 6 h: 176 kDa; 8 h: 168 kDa; 10 h: 158 kDa; 24 h: 130 kDa K: 10 h: 94 kDa; 12 h: 76 kDa; 3 × 4 h: 99.7 kDa; 24 h: 32 kDa	Soluble products obtained with both deacetylation methods; prolonged treatment generally reduced M_v_. SEM: Fibrous morphology of adult-derived material retained after deacetylation.	[13]
Dm: 0.5 M formic acid (1:10, *w*/*v*), RT, 1 h; Dp: 2 M NaOH (1:10), 80 °C, 2 h; Dec: 5% (*v*/*v*) H_2_O_2_ (1:20–30), 90 °C, 30–60 min; Da: 12 M NaOH (1:20), 100 °C, 4 h; Pur: 1% (*v*/*v*) acetic acid, RT, 48 h, filtered and precipitated	Gainesville diet	Cs/raw: Dec:3 ± 0.3%; NoDec: 2 ± 0.4% Cs/Ct: Dec: 41 ± 1%; NoDec: 27 ± 2%	Titration Dec: 93 ± 1.4%; NoDec: 91 ± 0.7%	M_v_ Dec: 36 ± 0.5 kDa; NoDec: 62 ± 0.1 kDa	CrI: Dec: 86%; NoDec: 79%; Films: both uniform; NoDec dark brown; Dec lighter but more pigmented than other bleached biomasses. SEM: Dec: less fibrillated, more homogeneous, porous.	[2]
Dm: 0.5 M formic acid, RT, 1 h; Dp: 2 M NaOH, 80 °C, 2 h; Dec: 5% H_2_O_2_, 90 °C, 60 min. Da: 12 M NaOH, 4 °C overnight;	NR	Cs/raw: Dec:2.0 ± 0.1%; NoDec: 1.0 ± 0.4%; Cs/Ct: Dec: 30 ± 0.5%; NoDec: 10 ± 2.3%;	Titration No Dec: 59 ± 2.9%; Dec: 61 ± 2.3%	M_v_ No Dec: 258 ± 28 kDa; Dec: 89 ± 6 kDa	No Dec CrI: 58% Dec CrI: 52% SEM: No Dec: greater structural complexity; Dec: smoother surface, less pronounced filamentous network.	[19]
Dp 5% NaOH, (1/15, *w*/*v*), 90 °C, 4 h Dm 4% HCl (1/15, *w*/*v*), RT, 12 h Dec: 1% KMnO_4_; (1/20, *w*/*v*), RT, 12 h Da: 70% NaOH, 80 °C, 8 h	NR	Cs/raw: 4.06%	Titration 71.9%	M_v_ 164.6 kDa	Protein 0.55%; ash 0.47% Solubility 94.1%	[23]
*Hermetia illucens*|Prepupae (Not by-products)|Chemical extraction						
Prepupae Dm: 0.5 M formic acid (1:10), RT, 30 min; Dp: 2 M NaOH, 80 °C, 2 h; Da: 12 M NaOH (60 g/800 mL), 90 °C, 3 h. Dec after Da: 5% (*v*/*v*) H_2_O_2_ (1:20), 90 °C, 15 min;	Standard diet: Poultry feed:water (40:60); Fruit diet: 75% fruit (apple:orange:banana, 1:1:1) + 25% Standart diet; Meat diet: 75% poultry meat + 25% Standart diet; Vegetable diet: 75% vegetables (celery, sweet pepper and potato) + 25% Standart diet; Mixed diet: 25% of each	NoDec Cs/raw: Standart diet: 4.69 ± 0.92% Fruit diet: 3.39 ± 0.42% Meat diet: 3.30 ± 0.28% Vegetable diet: 2.97 ± 0.67% Mixed diet: 2.40 ± 0.32% Cs/Ct: Standart diet: 67.22 ± 4.41% Fruit diet: 65.24 ± 5.09% Meat diet: 68.05 ± 2.19% Vegetable diet: 64.64 ± 2.31% Mixed diet: 64.91 ± 5.71%	NR	NR	R_AC_ values represent acetylation degree (scale of 0–1). Dec Cs: no significant differences between diets, R_AC_ ~0.3 NoDec Cs: All diets > Standart diet (+77.5% for fruit, +146.0% for vegetables, +149.6% for mixed, +169.5% for meat)	[25]
*Hermetia illucens*|Pupae (Not by-products)|Emerging solvent and intensified processes						
MA: 800 W (total process 24 min); Dm 3 M HCl (1:10), 50 °C; Dp 2 M NaOH (1:10), 80 °C, Da 12 M NaOH (1:20), 100 °C, 8 min Chemical extraction Dm: 3 M HCl (1:10), 50 °C, 2 h; Dp: 2 M NaOH (1:10), 80 °C, 2 h; Da: 12 M NaOH (1:20), 100 °C, 2 h	NR	Cs/raw Microwave: 3.89 ± 2.35% Chemical: 3.28 ± 0.35%	FTIR Microwave: 76.01% Chemical: 76.48%	NR	CrI: MA: 20.33%; Chemical: 37.76%; Mean particle size: MA: 1135.6 ± 964.9 nm. Chemical: bimodal, 539.4 ± 19.3 nm and 6840.0 ± 522.8 nm SEM: MA: Irregular porous/microfibrillar surface; relatively more homogeneous and less compact Chemical: Denser and more compact surface	[32]
*Tenebrio molitor*|Exuvia|Chemical extraction						
Dm: 5 g/100 mL 1 M HCl, 60 °C, 1.5 h; Dp: 100 mL 1 M NaOH, 100 °C, 20 h; Dec: water 95% ethanol:chloroform 4:2:1, 2 h Da: 60% (*w*/*v*) NaOH, 100 °C, 20 h.	NR	Cs/raw 6.26 ± 0.10% Cs/Ct: 74.93 ± 0.93%	Titration 76.32 ± 0.26% FTIR 81.06%	NR	RCI 65.3%; DTG: 245.8 °C; Residue: 4.14%; Moisture: 7.05%; Solubility: 71.69 ± 0.31%; Apparent viscosity: 215.72 ± 4.39 mPa.s SEM: showed a rough morphology	[5]
P1: Dm: 0.55 M HCl, RT, 15 + 30 min; Dec: 33% H_2_O_2_/NaHCO_3_ (9:1, *w*/*w*), RT, 30 min; Dp: 1 M NaOH, 80 °C, 4–5 × 45 min. P2: Dl: methanol:chloroform 2:1 (*v*/*v*), then same Dp. Da: at 120 °C mKOH:ethanol:monoethylene glycol 50:25:25; Durations: P1 24 h; P1 + Dl 6 h; P2 6/10/24 h	60% wheat bran + 40% poultry feed; fresh potato every 4 d LE1: Exuviae collected at 25 to 35 days LE2: Exuviae colected with >60 d	P2 6 h Cs/raw: LE1: 20.5% LE2: 21% Cs/Ct: LE1: 76% LE2: 75%	^1^H NMR LE1: P1, 24 h: 95%; P2, 6 h: 88.5%; 10 h: 91%; 24 h: 94%. LE2: P1, 24 h: 86%; P2, 6 h: 89%; 10 h: 90.5%; 24 h: 92%.	M_v_ LE1: P1, 24 h: 83 kDa P2; 6 h: 284 kDa; 10 h: 234 kDa; 24 h: 136 kDa LE2: P1, 24 h: 96 kDa P2; 6 h: 339 kDa; 10 h: 272 kDa; 24 h: 148 kDa	P1: residual organic impurities; soluble at 24 h; P2: reported as pure; soluble by 6 h SEM: P1 and P2, 24 h: original surface structure retained;	[4]
Dm: 2 N HCl, 20 °C, 3 h, Dp: 5% NaOH, 95 °C, 3 h; Da: 55% NaOH, 105 °C, 5 h.	NR	Cs/raw 7.8%	Titration 50.38%	NR	NR	[17]
Dm: 2 M HCl, RT, 3 h; Dp: 5% NaOH, 95 °C, 3 h; Da: 50% NaOH (*w*/*w*), 105 °C, 5 h; washed and dried (70 °C, 16 h). Pur: Cs dissolved in 1% acetic acid (*w*/*v*), filtered and precipitated.	Mixed grain-based substrate (ground corn, wheat bran, rice bran) and vegetables	Cs/raw: 7.41 ± 0.26%	Titration 72.27 ± 1.03%	M_v_ 612 kDa	TGA (unpurified): DTGmax 306.69 °C; residue ~29% at 600 °C.; Moisture 7.66 ± 0.54%; ash 0.33 ± 0.21%; Nitrogen: 6.59 ± 0.89%. SEM: Unpurified chitosan was rough, flaky and compact; the purified sample was smoother, porous and fibrous.	[21]
*Tenebrio molitor*|Exuvia|Enzymatic and microbial extraction						
Dp: Alcalase (2% enzyme/substrate), 3% (m/v) dispersion, pH 8, 50 °C, hydrolysis monitored to plateau; enzyme inactivated (90 °C, 15 min); centrifuged; Da: 40% NaOH (*w*/*v*), 90 °C, 8 h	Corn/wheat/rice bran and vegetables;	Cs/raw: 31.9% Cs/Ct: 45.1%	Titration 53.9%	NR	TGA DTGmax 314.7 °C; Moisture 4.2 ± 0.1%; ash 3.7 ± 0.1%; protein 5.4 ± 0.2%.; Solubility 40.3 ± 0.6%.	[27]
*Tenebrio molitor*|Exuvia|Chemical extraction and autoclave-assisted deacetylation						
M1: Dm: 5% HCl (1:6), 40 °C, 2 h; Dp 3% NaOH (1:6), 60 °C, 4 h; Da 50% NaOH (sample:NaOH:water 1:6:2), 80 °C, 2 h. M2: M1 without Dm. M3: Dp: 5% NaOH (1:6), 70 °C, 3.5 h; Da (AA) 50% NaOH, 121 °C/15 psi, 30 min.	NR.	Cs/raw M1 6.77 ± 0.38%; M2 8.27 ± 0.60%; M3 8.87 ± 0.35%.	Titration: M1 42.62 ± 0.38%; M2 25.25 ± 1.2%; M3 55.62 ± 0.79% M1 and M2 partially deacetylated materials reported as chitosan by the authors	M_v_ M1 71 ± 0.05 kDa; M2 97 ± 1.17 kDa; M3 116 ± 2.53 kDa.	Solubility: M1 12.74 ± 0.82%; M2 9.81 ± 0.81%; M3 24.13 ± 2.07%. Films, swelling (SD%): M1 (1.5%) 5.07 ± 0.11; M1 (1%) 7.77 ± 0.76; M2 (1%) 13.3 ± 1.2; Film weight loss: M1 (1.5%) 2.46 ± 0.43%; M1 (1%) 4.88 ± 0.41%	[28]
*Tenebrio molitor*|Dead adults|Chemical extraction						
P1: Dm: 0.55 M HCl, RT, 15 + 30 min; Dec: 33% H_2_O_2_/NaHCO_3_ (9:1, *w*/*w*), RT, 30 min; Dp: 1 M NaOH, 80 °C, 4–5 × 45 min. P2: Dl: methanol:chloroform 2:1 (*v*/*v*), then same Dp. Da: Broussignac (B): 120 °C: KOH:ethanol:MEG 50:25:25, 24 h; Kurita (K): 50% NaOH, 24 h at 120 °C; Listed durations: P1 24 h; P1 + Dl 6 h; P2 6/10/24 h	60% wheat bran + 40% poultry feed; fresh potato every 4 d	Cs/raw: B/P1 4 h: 19.5% B/P2 4 h: 24% Cs/Ct: B/P1 4 h: 78% B/P2 4 h: 80% K/P1 10 h: 70–75%	^1^H NMR B/P1; 4 h: 89%; 6 h: 91%; 8 h: 92%; 10 h: 94%; 24 h: 88% B/P2; 4 h: 91.5%; 6 h: 92%; 8 h: 93%; 10 h: 94%; 24 h: 95%; K/P1:; 10 h: 95%; 12 h: 96%; 3 × 4 h: 97%; 24 h: 97%	M_v_ B/P1: 4 h: 263 kDa; 6 h: 229 kDa; 8 h: 219 kDa; 10 h: 200 kDa; 24 h: 137.5 kDa; B/P2: 4 h: 284 kDa; 6 h: 263 kDa; 8 h: 243 kDa; 10 h: 221 kDa; 24 h: 153 kDa; K/P1: 10 h: 132 kDa; 12 h: 121 kDa; 3 × 4 h: 126 kDa; 24 h: 91 kDa.	B/P1 and B/P2: pure; fully soluble by 4 h.; K/P1: fully soluble by 10 h SEM: B/P1 and B/P2, 24 h: original surface structure retained;	[4]
Dm: 5 g/100 mL 1 M HCl, 60 °C, 1.5 h; Dp: 100 mL 1 M NaOH, 100 °C, 20 h; Dec: water 95% ethanol:chloroform 4:2:1, 2 h Da: 60% (*w*/*v*) NaOH, 100 °C, 20 h.	NR	Cs/raw 6.52 ± 0.06% Cs/Ct: 78.96 ± 0.45%	Titration 89.21 ± 0.96% FTIR 87.29%	NR	RCI 67.6%; DTG: 326.8 °C; Residue: 30.91%; Moisture: 6.89%; Solubility: 94.41 ± 0.95%; Apparent viscosity: 216.68 ± 2.43 mPa.s SEM: the sample exhibited pores in its structure	[5]
*Tenebrio molitor*|Dead adults|Enzymatic and microbial extraction						
Dp: 1:10 (*w*/*v*) in water; 2% Alcalase^®^, 70 °C; time, 6 h, pH 10 Da: 60% NaOH 1:10 (*w*/*v*), 100 °C, 24 h Dec: 30% H_2_O_2_, 90 °C, 30 min	NR	NR	FTIR 85%	NR	TGA: polymer decomposition ~230–400 °C; stable residue ~2% above 600 °C. SEM (×300): homogeneous structure with irregular aggregates. Water-binding capacity: 773 ± 19%; fat-binding capacity: 715 ± 12%. Fully soluble at 1% (*w*/*v*) in 0.5% (*v*/*v*) acetic acid.	[16]
*Tenebrio molitor*|Adults (not by-products)|Chemical extraction						
M1: Dm: 2 M HCl, 65–75 °C, 2 h; Dp: 2 M NaOH, 80–90 °C, 16 h; Dec: chloroform:methanol:water 1:2:4, 1 h. M2: 3% NaClO, 100 °C, Dm: 1 M HCl, 75 °C, 15 min; Dp: 1 M NaOH, 100 °C, 20 min. M1 and M2: Da: 50% (*w*/*v*) NaOH (1:20), 100 °C, 3 h.	NR	Cs/Ct M1: 78.26% M2: 76.43%	FTIR Method 1: 75.84%; Method 2: 72.6%	NR	Solubility: M1 95.3 ± 0.4%; M2 92.3 ± 0.2%;	[26]
*Tenebrio molitor*|Adults (not by-products)|Chemical extraction followed by microwave-assisted depolymerisation						
Dm: 2 M HCl, 50 °C, 24 h; Dp: 2 M NaOH, 50 °C, 24 h; Purification: chloroform:methanol:water 1:2:4; Da: 6.5 g/150 mL 60% NaOH, 120 °C, 2 h, Depol: 1% (*w*/*v*) CS/1% (*v*/*v*) acetic acid, microwave 800 W, 6/18/30 min in cycles (T6/T18/T30); T0: unirradiated, freeze-dried control.	NR	NR	Titration TM: 51.32 ± 1.01%; T0: 50.98 ± 1.34%; T6: 52.33 ± 4.35%; T18: 50.31 ± 1.16%; T30: 50.72 ± 3.27%	M_v_ TM: 492.49 ± 45.37 kDa; T0: 419.02 ± 23.40 kDa; T6: 378.17 ± 9.32 kDa; T18: 311.26 ± 18.49 kDa; T30: 328.90 ± 2.72 kDa	CrI: TM: 73.84 ± 0.93%; T0 70.10 ± 1.44%; T6: 71.09 ± 0.99%; T18: 66.26 ± 2.10%; T30: 69.10 ± 1.88%; DTG:TM: 302.27 °C; T0: 297.71 °C; T6: 297.88 °C; T18: 297.46 °C; T30: 297.23 °C; Residue: TM: 0.06%; T0: 1.23%; T6: 0.00%; T18: 0.00%; T30: 0.00%; Consistency index: TM: 7.42 ± 0.43 Pa.sn; T0: 3.46 ± 0.16 Pa.sn; T6: 2.25 ± 0.16 Pa.sn; T18: 2.72 ± 0.04 Pa.sn; T30: 1.56 ± 0.10 Pa.sn TM: moderate brightness (natural pigments); T0: darker, much lower brightness; T6: brighter than T0; T18: maximum brightness; T30: slightly lower than T18	[34]

CS/Cs, chitosan; Ct, chitin; Dm, demineralisation; Dp, deproteinisation; Dl, delipidisation; Da, deacetylation; Dec, decolourisation or decolourised sample; NoDec, non-decolourised sample; Pur, purification or purified sample; Depol, depolymerisation; Het, heterogeneous deacetylation; Hom, homogeneous deacetylation; B, Broussignac method; K, Kurita method; AA, autoclave-assisted; MA, microwave-assisted; DES, deep eutectic solvent; MEG, monoethylene glycol; STPP, sodium tripolyphosphate; DDA, degree of deacetylation; MW, molecular weight; M_v_, viscosity-average molecular weight; FTIR, Fourier-transform infrared spectroscopy; ^1^H NMR, proton nuclear magnetic resonance spectroscopy; SEM, scanning electron microscopy; DLS, dynamic light scattering; CrI, crystallinity index; RCI, relative crystallinity index; TGA, thermogravimetric analysis; DTG, derivative thermogravimetry; DTGmax, temperature of maximum mass-loss rate; R_AC_, Relative Absorbance Ratio reported in [25]; SD%, swelling degree; RT, room temperature; NR, not reported. Materials labelled “not byproducts” provide comparative evidence from other insect biomass sources.

**Table 2:** Bioactivity of chitosan and nanochitosan derived from *Hermetia illucens* and *Tenebrio molitor*: extraction conditions, material characteristics, assay conditions and main outcomes. Studies using non-by-product biomass are included for comparison and explicitly identified.

Extraction Conditions	DDA/MW	Bioactivity	Concentrations	Results	Ref.
*Hermetia illucens*|Puparia|Chemical extraction					
Dm: 0.5 M formic acid, RT, 1 h; Dp: 2 M NaOH, 80 °C, 2 h; Dec: 5% H_2_O_2_, 90 °C, 1 h; Da: 12 M NaOH, 100 °C, 4 h; Pur: 1% acetic acid, filtration and precipitation.	NR	Antimicrobial: agar diffusion and microdilution against *E. coli* and *M. flavus*.	Cs: 1.25, 0.6, 0.3 and 0.15 mg/mL. Comparators: acetic acid solution and commercial Cs.	Zones across tested concentrations: *E. coli*, 8–9 mm, Dec/NoDec; *M. flavus*, Dec 7–10 mm, NoDec 7–9 mm. MIC (*E. coli*/*M. flavus*, mg/mL): Dec 0.30/0.15; NoDec 0.15/0.30. MIC interpretation limited by solvent inhibition.	[18]
Dm: 0.5 M formic acid (1:10); Dp: 2 M NaOH (1:10), 80 °C, 2 h; Dec subset: 5% H_2_O_2_ (1:20–30), 90 °C, 30–60 min. Da: Het, 12 M NaOH (1:20), 100 °C, 4 h; Hom, 12 M NaOH, 4 °C overnight; ice added, soluble fraction incubated at RT, 24–48 h; HCl precipitation.	NR	Indirect response: HBD-2 gene expression by qPCR in LPS-stimulated HaCaT cells; Cs 0.5 mg/mL, 6/24 h. Viability: 97–100% at 24 h across tested samples. Direct antimicrobial activity: microdilution and CFU counts against *E. faecalis*, *S. epidermidis* and *S. agalactiae*.	Indirect: CS 0.5 mg/mL; LPS 20 µg/mL; 6/24 h; viability at 24 h. Direct: CS 0.0015–0.5 mg/mL; CFU assessment at 0.5 mg/mL, 24 h, 37 °C. Comparators: bacterial control and commercial CS for direct assays.	HBD-2 expression: Hom NoDec > Dec at 6/24 h; Het Dec strongest among heterogeneous samples, comparable to commercial CS. Direct, Hom NoDec: *E. faecalis* 2 × 10^9^ versus 1 × 10^11^ CFU/mL for bacterial control/commercial CS; *S. epidermidis*, 3-log reduction versus control; *S. agalactiae*, 3.3 × 10^8^ CFU/mL, significantly below control/commercial CS. Hom Dec and Het: limited or non-significant effects, depending on sample and organism.	[20]
Dm: 0.5 M formic acid, RT, 1 h; Dp: 2 M NaOH, 80 °C, 2 h; Dec subset: 5% H_2_O_2_, 90 °C, 1 h; Da (Hom): 12 M NaOH, 4 °C overnight; ice added, soluble fraction incubated at RT, 24–48 h; HCl precipitation; Pur: 1% acetic acid dissolution and reprecipitation.	DDA: Dec 72%; NoDec 62%. M_v_: Dec 115 kDa; NoDec 285 kDa.	Antioxidant: DPPH scavenging, IC_50_. Antimicrobial: agar diffusion and microdilution against *E. coli*/*M. flavus*.	DPPH: 0.156–5 mg/mL; 30 min, dark, RT. Antimicrobial: 0.15–1.25 mg/mL; CS prepared in 1% acetic acid; microdilution, 37 °C, 24 h; acetic acid and bacterial controls.	DPPH IC_50_ (mg/mL): Dec 7.9 ± 0.3; NoDec 12.1 ± 1.0; greater scavenging with Dec. MIC (mg/mL): *E. coli*, 0.15 for both; *M. flavus*, Dec 0.15, NoDec 0.30.	[19]
Dp: 5% NaOH (1:15), 90 °C, 4 h; Dm: 4% HCl (1:15), RT, 12 h; Dec: 1% KMnO_4_ (1:20), RT, 12 h; Da: 70% NaOH, 80 °C, 8 h.	DDA: 72.3%. MW: 194.9 kDa, GPC; average type unspecified.	Antimicrobial: disc diffusion against *B. subtilis* and *V. alginolyticus*.	*B. subtilis*: 0.25, 0.50, 0.75 and 1.00% (*w*/*v*); *V. alginolyticus*: 1.00, 1.50, 1.75 and 2.00% (*w*/*v*). CS in 1% acetic acid; 40 µL/6 mm disc; 24 h, RT. Controls: 1% acetic acid and tetracycline, 30 µg.	Reported inhibition zones: *B. subtilis* 4.67–6.33 mm; *V. alginolyticus* 3.70–4.40 mm	[23]
Dm: 1 M HCl, 10 mL/g, RT, 1 h; Dp: 1 M NaOH, 25 mL/g, 80 °C, 13 × 1 h; Da: 50% NaOH (1:30), 90 °C, 10 h. Nanoformulation: 3% CS in 2% acetic acid, 24 h; STPP ionic gelation, 1000 rpm, 45 min; pH adjusted to 5.	Nanochitosan DDA: 85.3%; MW: NR.	Antimicrobial: agar diffusion; microdilution MIC and plating MBC against *B. subtilis*/*E. cloacae*.	Diffusion: 80–1280 µg/mL, twofold dilutions, 5 µL/disc. Microdilution: 10–1280 µg/mL in MH broth with 1% acetic acid; 37 °C, 24 h. Controls: water and 1% acetic acid for diffusion; 1% acetic acid for microdilution.	Nanochitosan: zones increased from 6.1 to 13.1 mm (*E. cloacae*) and from 6.1 to 12.4 mm (*B. subtilis*), with no significant difference between 640 and 1280 µg/mL. MIC/MBC: 320/640 and 160/320 µg/mL, respectively. Slight, unquantified solvent inhibition.	[24]
*Hermetia illucens*|Puparia|Emerging solvent and intensified processes					
Dl: petroleum ether, Soxhlet, 55 °C, 6 h. DES: choline chloride-lactic acid, 92 °C, 5.6 h, 20 mL/g. Dec: 20% H_2_O_2_, pH 7.5. Da: NaOH (1:30); Cts1: 60-mesh, 33%, 90 °C, 3 h; Cts2: 80-mesh, 33%, 90 °C, 3 h; Cts3: 80-mesh, 42%, 120 °C, 4.5 h.	DDA, titration: Cts1 36.3%; Cts2 41.85%; Cts3 65.3%.	Haemostasis: BCI and experimental rat femoral-vessel injury. Associated assays: PT/APTT, platelet adhesion and protein adsorption. Haemolysis; lysozyme-mediated degradation.	BCI: 6 mg/100 µL blood + 10 µL 0.2 M CaCl_2_, 60 s. Haemolysis: 6 mg/mL, 3 h. Cts2 degradation: 15 mg/mL in PBS + 10 µg/mL lysozyme, 2–12 d; all at 37 °C. Rat model: 50 mg topically; Cts2/Cts3/Celox, *n* = 3/group.	Cts2: lowest BCI; haemolysis < 5% under tested conditions; ~40% mass retained at day 12. Rat blood loss: 285 ± 37 mg versus Celox 462 ± 43 mg and Cts3 535 ± 56 mg; haemostasis: 2.17 ± 0.24 versus ~3 min. PT/APTT like untreated plasma; increased platelet adhesion and preferential fibrinogen adsorption.	[33]
*Hermetia illucens*|Dead adults|Chemical extraction					
Dm: 0.5 M formic acid, RT, 1 h; Dp: 2 M NaOH, 80 °C, 2 h; Dec subset: 5% H_2_O_2_, 90 °C, 1 h; Da: 12 M NaOH, 100 °C, 4 h; Pur: 1% acetic acid, filtration and precipitation.	NR	Antimicrobial: agar diffusion and microdilution against *E. coli*/*M. flavus*.	CS: 1.25, 0.6, 0.3 and 0.15 mg/mL. Comparators: acetic acid solution and commercial CS.	Zones: 8–10 mm against both bacteria across tested concentrations; similar Dec/NoDec responses. MIC (*E. coli*/*M. flavus*, mg/mL): Dec 0.30/0.15; NoDec not determined. MIC interpretation limited by solvent inhibition.	[18]
Dm: 0.5 M formic acid, RT, 1 h; Dp: 2 M NaOH, 80 °C, 2 h; Dec subset: 5% H_2_O_2_, 90 °C, 1 h; Da (Hom): 12 M NaOH, 4 °C overnight; ice added, soluble fraction incubated at RT, 24–48 h; HCl precipitation; Pur: 1% acetic acid dissolution and reprecipitation.	DDA: Dec 61%; NoDec 59%. M_v_: Dec 89 kDa; NoDec 258 kDa.	Antioxidant: DPPH scavenging, IC_50_. Antimicrobial: agar diffusion and microdilution against *E. coli*/*M. flavus*.	DPPH: 0.156–5 mg/mL; 30 min, dark, RT. Antimicrobial: 0.15–1.25 mg/mL; CS prepared in 1% acetic acid; microdilution, 37 °C, 24 h; acetic acid and bacterial controls.	DPPH IC_50_ (mg/mL): Dec 7.5 ± 0.5; NoDec 17.8 ± 0.2; greater scavenging with Dec. MIC: 0.15 mg/mL for both bacteria and treatments.	[19]
Dp: 5% NaOH (1:15), 90 °C, 4 h; Dm: 4% HCl (1:15), RT, 12 h; Dec: 1% KMnO_4_ (1:20), RT, 12 h; Da: 70% NaOH, 80 °C, 8 h.	DDA: 71.9%. MW: 164.6 kDa, GPC; average type unspecified.	Antimicrobial: disc diffusion against *B. subtilis* and *V. alginolyticus*.	*B. subtilis*: 0.25, 0.50, 0.75 and 1.00% (*w*/*v*); *V. alginolyticus*: 1.00, 1.50, 1.75 and 2.00% (*w*/*v*). CS in 1% acetic acid; 40 µL/6 mm disc; 24 h, RT. Controls: 1% acetic acid and tetracycline, 30 µg.	Reported inhibition zones: *B. subtilis* 4.33–6.00 mm; *V. alginolyticus* 2.34–4.02 mm.	[23]
*Hermetia illucens*|Adult (not by-product)|Chemical extraction					
Dm: 0.5 M formic acid (1:10); Dp: 2 M NaOH (1:10), 80 °C, 2 h; Dec subset: 5% H_2_O_2_ (1:20–30), 90 °C, 30–60 min. Da: Het, 12 M NaOH (1:20), 100 °C, 4 h; Hom, 12 M NaOH, 4 °C overnight; ice added, soluble fraction incubated at RT, 24–48 h; HCl precipitation.	NR	Indirect response: HBD-2 gene expression by qPCR in LPS-stimulated HaCaT cells; Cs 0.5 mg/mL, 6/24 h. Viability: 97–100% at 24 h across tested samples. Direct antimicrobial activity: microdilution and CFU counts against *E. faecalis*, *S. epidermidis* and *S. agalactiae*.	Indirect: CS 0.5 mg/mL; LPS 20 µg/mL; 6/24 h; viability at 24 h. Direct: CS 0.0015–0.5 mg/mL; CFU assessment at 0.5 mg/mL, 24 h, 37 °C. Comparators: bacterial control and commercial CS for direct assays.	HBD-2 expression: Hom NoDec > Dec; Het Dec induced expression; Het NoDec weaker than puparia-derived variants. Direct effects limited overall: Hom Dec, *E. faecalis* 2 × 10^10^ CFU/mL, below control; Hom/Het Dec slightly reduced *S. epidermidis* absorbance.	[20]
*Tenebrio molitor*|Exuvia|Chemical extraction					
Dm: 2 M HCl, RT, 3 h; Dp: 5% NaOH, 95 °C, 3 h; Da: 50% NaOH (*w*/*w*), 105 °C, 5 h; Pur: dissolution in 1% acetic acid, filtration and precipitation.	DDA: 72.27%. M_v_: 612 kDa.	Antioxidant: DPPH radical-scavenging assay.	Cs solutions: 2, 5 and 10 mg/mL in 1% acetic acid; 200 µL sample + 1000 µL DPPH; 30 min, dark, RT.	DPPH scavenging: 11.96 ± 0.49%, 32.51 ± 1.39% and 62.75 ± 2.91%, at 2, 5 and 10 mg/mL, respectively; reported IC_50_ 7.92 mg/mL. Concentrations refer to added Cs solutions.	[21]
*Tenebrio molitor*|Exuvia|Chemical extraction and autoclave-assisted deacetylation					
M1: Dm, 5% HCl (1:6), 40 °C, 2 h; Dp, 3% NaOH (1:6), 60 °C, 4 h; Da, 50% NaOH (sample:NaOH:water 1:6:2), 80 °C, 2 h. M2: M1 without Dm. M3: Dp, 5% NaOH (1:6), 70 °C, 3.5 h; Da, 50% NaOH, autoclave, 121 °C/15 psi, 30 min.	DDA: M1 42.62%; M2 25.25%; M3 55.62%. M_v_: M1 71; M2 97; M3 116 kDa.	Antimicrobial: viable counts and percentage inhibition against *E. coli*, *S. aureus*, *E. aerogenes*, *P. aeruginosa* and *Salmonella* spp.	1% Cs in 0.5% acetic acid, adjusted to pH 4.8; 5 mL added to inoculated TSB; 35 °C, 24/48 h.	M1/M2: high reported inhibition of *E. aerogenes*/*P. aeruginosa* at 24/48 h; other responses generally improved at 48 h. M3: greater activity at 48 h, particularly against *E. aerogenes*/*Salmonella*; *S. aureus* least susceptible.	[28]
*Tenebrio molitor*|Adults (not by-products)|Chemical extraction					
M1: Dm, 2 M HCl, 65–75 °C, 2 h; Dp, 2 M NaOH, 80–90 °C, 16 h; Dec, chloroform:methanol:water (1:2:4), 1 h. M2: 3% NaClO, 100 °C; Dm, 1 M HCl, 75 °C, 15 min; Dp, 1 M NaOH, 100 °C, 20 min. Both: Da, 50% NaOH (*w*/*v*; 1:20), 100 °C, 3 h.	DDA: M1 75.84%; M2 72.6%. MW: NR.	Antimicrobial: disc diffusion against *P. aeruginosa*.	CS: 4% and 8% in 1% acetic acid; autoclaved, 121 °C, 20 min; 20 µL/6 mm disc; 37 °C, 24 h; duplicate assays. Comparators: commercial CS, ampicillin (1 mg/mL), PBS and acetic acid.	At 8%, reported inhibition zone: 4 mm versus commercial CS 2 mm and ampicillin 10 mm; no inhibition at 4% or with acetic acid. Results not resolved by extraction method (M1/M2).	[26]
*Tenebrio molitor*|Adults (not by-products)|Chemical extraction followed by microwave-assisted depolymerisation					
Dm/Dp: 2 M HCl/NaOH, each 50 °C, 24 h; Pur: chloroform:methanol:water (1:2:4); Da: 6.5 g/150 mL 60% NaOH, 120 °C, 2 h. Depol: 1% CS in 1% acetic acid, microwave 800 W, cyclic 6/18/30 min (T6/T18/T30); T0: non-irradiated, freeze-dried control.	M_v_ (kDa): TM 492.49; T0 419.02; T6 378.17; T18 311.26; T30 328.90.	Antioxidant: DPPH scavenging, IC_50_. Antimicrobial: MIC/MBC against *L. monocytogenes*, *S. aureus*, *E. coli* and *Salmonella* spp.	DPPH: CS solutions 0.25–5 mg/mL in 0.5% (*v*/*v*) acetic acid. Antimicrobial stock: 5 mg/mL in 0.5% acetic acid, pH adjusted to 5.9; reported serial dilution range 0.002–0.5% in 0.9%	DPPH IC_50_: TM 0.30 ± 0.07; T0 0.71 ± 0.04; T6 0.52 ± 0.02; T18 0.79 ± 0.03; T30 0.71 ± 0.04. MIC/MBC (mg/mL): *L. monocytogenes*: TM 0.16/0.16; T0/T6 0.04/0.16; T18/T30 0.04/0.08. *E. coli*: TM/T0/T6 1.25/1.25; T18/T30 0.63/0.63. *Salmonella* spp.: T0 2.50/2.50; all others 2.50/5.00. *S. aureus*: TM 0.08/0.63; T6/T18/T30 0.08/0.16; TM had the lowest numerical DPPH IC_50_; antimicrobial responses varied by organism and treatment.	[34]

Cs, chitosan; Dm, demineralisation; Dp, deproteinisation; Dl, delipidisation; Da, deacetylation; Dec, decolourisation or decolourised sample; NoDec, non-decolourised sample; Pur, purification; Depol, depolymerisation; Het, heterogeneous deacetylation; Hom, homogeneous deacetylation; DES, deep eutectic solvent; STPP, sodium tripolyphosphate; DDA, degree of deacetylation; MW, molecular weight; M_v_, viscosity-average molecular weight; GPC, gel permeation chromatography; DPPH, 2,2-diphenyl-1-picrylhydrazyl; IC_50_, concentration required to scavenge 50% of DPPH radicals; MIC, minimum inhibitory concentration; MBC, minimum bactericidal concentration; CFU, colony-forming units; HBD-2, human β-defensin-2; qPCR, quantitative polymerase chain reaction; LPS, lipopolysaccharide; HaCaT, immortalised human keratinocyte cell line; BCI, blood coagulation index; PT, prothrombin time; APTT, activated partial thromboplastin time; PBS, phosphate-buffered saline; MH, Mueller-Hinton; TSB, tryptic soy broth; RT, room temperature; NR, not reported; *w*/*v*, weight/volume; *w*/*w*, weight/weight; *v*/*v*, volume/volume.

**Table 3:** Chitosan-based biomaterials for veterinary applications: material origin, formulations, study designs, comparators and outcomes across in vitro studies, experimental animal models and veterinary clinical studies.

Application	Chitosan	Formulation	Study Design	Comparator	Duration	Main Outcomes and Limitations	Ref.
Wound management	Medical-grade Cs ChitoHeal^®^; DDA 85–90%	Non-woven cellulose dressing containing 2% (*w*/*w*) Cs; topical application; bandage changed every 48 h	Experimental animal study: 12 horses; 24 paired, randomised distal-limb wounds, 12 wounds/treatment	Sterile gauze under the same bandaging protocol	42 d	Day-42 wound area remaining: 6.2 ± 1.7% vs. 19.8 ± 2.8%; time to 50% closure: 16.4 ± 2.1 vs. 23.7 ± 3.3 d (both *p* < 0.001; mean ± SD). Day-28 granulation coverage: 93.6% vs. 72.4%. No Cs-related adverse events reported. Standardised experimental wounds.	[71]
Wound management	Shrimp-shell-waste Cs;	Milled Cs powder; topical application once daily after saline cleansing; dose NR; gauze/bandage for 7 d, then uncovered for 21 d	Veterinary case report: one 3-year-old female German shepherd with infected traumatic wounds; concurrent antibiotics and supportive care	NR	28 d of Cs treatment	Clotting, exudate absorption, granulation and healing progression reported over 4 weeks.	[76]
Wound management	Shrimp-shell Cs; reported DDA 92%	Cs lactate powder applied once to the right buccal wound.	Experimental animal study: 9 male dogs; paired 1 cm-diameter, 2 mm-deep buccal wounds; 3 dogs evaluated per time point	Contralateral wound treated with Cs -free gauze	5, 10 and 15 d	Bleeding time: 138 (74–345) vs. 309 (141–458) s, median (IQR), *p* = 0.139; wound-area reduction: 69.63 ± 22.16% vs. 52.66 ± 32.10%, *p* = 0.210. Lower acute inflammation and neovascularisation; higher collagenisation, granulation and re-epithelialisation (*p* = 0.025–0.046).	[78]
Wound management	Sigma-Aldrich Cs; DDA 75–85%; medium MW	Freeze-dried Cs/GPTMS/DSP membranes. In vivo: gentamicin-sulphate-loaded membranes, prepared using 3.5 mg/mL loading solution; 2 mm thickness; implanted dose NR. Separate in vitro formulations contained 5, 10 or 15% AgNPs (*w*/*w* relative to Cs)	In vitro characterisation, release and antimicrobial testing; preliminary veterinary case series: 12 Hermann’s tortoises (*Testudo hermanni*) with shell injuries	In vitro: unloaded Cs/GPTMS/DSP membranes. In vivo: no concurrent control reported	Implantation: 21 d; in vitro release/degradation: up to 56 d	All tortoises were reported to recover; mould developed in 2 cases, requiring membrane removal and replacement. Gentamicin release: approximately 70% by 24 h, followed by approximately 0.55%/d. Antibacterial responses depended on loading and organism.	[77]
Wound management	NR	Autologous PRP alone, PRP/Cs nanoparticles or PRP/Cs -capped Ag nanoparticles. Single intradermal administration at four wound-margin sites: PRP alone, 1 mL; combinations, 1 mL PRP + 1 mL nanoparticle preparation.	Randomised experimental animal study: 24 male mongrel dogs; 4 groups, *n* = 6/group; one 2 × 2 cm wound/dog; blinded macroscopic assessment	Saline; PRP alone; PRP/Cs nanoparticles	21 d; histology and bacterial assessments on days 7, 14 and 21	PRP/Cs/Ag showed the greatest reported contraction and lowest bacterial burden; higher collagen deposition than other groups on days 14/21 and higher EGF expression. PRP/Cs showed higher α-SMA expression. Key quantitative outcomes are graphical.	[74]
Wound management	Commercial Cs	Lyophilised Cs or Cs -Na-free bioactive-glass scaffolds. Implantation through an 8 mm incision; diabetic wounds 1 × 1 cm; scaffold dose NR. Toxicity: aqueous/PBS extracts IV and oil extracts IP, 50 mL/kg	Experimental animal studies: female Sprague-Dawley rats for implantation (*n* = 40), sensitisation (*n* = 20) and acute toxicity (*n* = 20). Diabetic wounds: male Wistar rats, one rat/group, three groups	Implant/safety assays: appropriate scaffold-free or extraction-medium controls. Diabetic wounds: untreated control and Cs -only scaffold	Implantation: 1–4 weeks; sensitisation: staged induction/challenge through day 14, then 24–72 h assessments; acute toxicity: 72 h; wounds: 14 d	No sensitisation or acute systemic/organ toxicity reported under tested conditions. Cs -bioactive-glass wound showed complete closure by day 14. Wound experiment reports *n* = 1/group; efficacy and between-animal variability cannot be established.	[81]
Wound management	Commercial Cs, Vitex Pharmaceuticals, Australia	Cs powder beneath a 1 × 1 cm autologous skin graft transferred from forelimb to hindlimb; dose NR; gauze changed every 2 d; postoperative medication	Randomised experimental animal study: 18 adult female rabbits allocated to two groups	Same grafting procedure without Cs	21 d	Healing occurred in both groups. More follicles and thinner scar tissue were described qualitatively in Cs -treated grafts.	[80]
Wound management	NR	Cs powder, 1 g topically on days 1, 3 and 7, then weekly until recovery; lesion care and bandaging	Veterinary clinical study: 41 working equines, 37 donkeys and 4 horses; Cs *n* = 21, control *n* = 20; random allocation reported	Conventional cleansing, antiseptic/povidone-iodine treatment and bandaging every 2 d	Until recovery; follow-up extended to 32 weeks in one Cs -treated case; control-group horn development reported through 23 weeks	At 3 weeks, lameness score 0–1 in 18/21 vs. 4/20. Thin cornified layer covering the wound reported at 5 vs. 8–12 weeks. Bleeding absent by day 3 in Cs -treated animals versus persisting >1 week in controls; this is a follow-up observation, not an immediate haemostasis-time measurement.	[75]
Haemostasis and bleeding control	Commercial Cs	Cs fibre dressing (CF): 10 × 10 cm, 1.55–31 mg/cm^2^; Cs sponge: dimensions/mass NR. Applied after 45 s of bleeding; 5 min compression, with replacement/additional compression if required; IV resuscitation	Experimental animal study: 10 castrated male Yorkshire-cross pigs, 34–37 kg; 6 mm femoral arteriotomy; CF *n* = 4, sponge *n* = 3, gauze *n* = 3	Surgical gauze	Up to 180 min or death; limb movement tested at endpoint	Haemostasis: CF 3.25 ± 1.26 min; sponge 2.67 ± 0.58 min. Gauze: >20 min. Survival: 4/4, 3/3 and 0/3; resuscitation fluid: 30.55, 31.58 and 188.32 mL/kg, respectively (overall *p* < 0.001). No rebleeding after limb movement in Cs-treated animals.	[73]
Haemostasis and bleeding control	Commercial Cs, Macklin; DDA ≥ 95%;	Catechol-modified Cs (DCS) cross-linked with aldehyde-functionalised PEG/sebacic-acid polymer (PEGSH); five formulations. DCS/PEGSH1.0 selected for animal haemostasis; topical dose 10 mg. Haemolysis: 0.65–10 mg/mL	In vitro blood/cell assays and experimental rodent liver, tail and foot bleeding models; *n* = 3 animals weighing 150–170 g	Commercial haemostatic sponge containing Cs and gelatin; untreated/no-hydrogel controls; assay-specific controls	BCI endpoint: 5 min; cell assays: up to 3 d; degradation: up to 5 weeks; acute bleeding endpoints	Hydrogel clotting times < 50 s vs. 760 ± 13 s untreated and 288 ± 10 s commercial sponge. DCS/PEGSH1.0 reduced liver-model blood loss by approximately 90% vs. untreated control and showed >95% lethality against *E. coli*/*S. aureus*. Haemolysis < 5%; L929 viability > 75% under tested conditions.	[87]
Scaffolds for tissue regeneration and surgical repair	NR	Lyophilised sodium hyaluronate: Cs (HA: Cs) or pectin: Cs (P: Cs) scaffolds, both 2:1 mass ratio, inserted into canals after induction of a blood clot	Experimental animal study: 4 male beagles; 96 roots, block-randomised. Four intervention groups: 19 roots each initially; 20 additional control roots.	Blood clot alone; MTA apexification; untreated infected/pulp controls and normal roots	13 weeks	Periapical-lesion resolution: HA: Cs 47%, P: Cs 58%, blood clot 79%, apexification 94%. Cs scaffolds did not improve mineralised-tissue formation vs. blood clot. Residual scaffold/inflammation and resorption were observed; newly formed tissue did not reproduce normal pulp.	[79]
Scaffolds for tissue regeneration and surgical repair	Crab-derived Cs, DVR Pharm; estimated DDA 65% reported from spectral calculation;	Freeze-dried Cs; collagen: Cs composites, 1:1 or 3:1 mass ratios; collagen: Cs:clotrimazole, 1:1:0.1. Cell exposure: 10–500 µg/mL; diffusion assay: 6 × 6 mm material squares	In vitro study: agar diffusion against bacterial/fungal strains and HUVEC viability; antimicrobial assays in triplicate	Collagen, Cs and unloaded composites; antibiotic/antifungal references; cell-culture control	Diffusion: 18 h, 37 °C for bacteria/25 °C for fungi; HUVEC: 24, 48 and 72 h	Clotrimazole composite: zones 23.9 mm against *C. albicans*, 18.67 mm against *S. aureus* and 21.8 mm against MRSA; no *P. aeruginosa* activity. HUVEC viability was formulation-, dose- and time-dependent: Cs approximately 44% at 10 µg/mL/24 h; collagen: Cs 3:1, 50% at 500 µg/mL/48 h; collagen: Cs 1:1, 57.8% at 500 µg/mL/72 h. Other 72 h values generally >93%.	[88]
Drug, vaccine and biological delivery systems	Koyo Cs DAC-100; DDA 85%; MW 235 kDa,	3% (*w*/*v*) Cs gel in 2% acetic acid; MN and CHX, each 2% (*w*/*v*), with TE 0.4:0.025% (*w*/*v*). CHX incorporated as a solution or in TPP-cross-linked Cs nanoparticles. Cell assays: antimicrobial concentrations 0.625–2.5 µg/mL	In vitro study: bacterial/fungal microdilution, release testing and L929 fibroblast viability; antimicrobial assays in triplicate; cell-viability figure reports *n* = 5	Individual drugs, drug combinations, unloaded Cs gel, formulation controls and microbial growth/medium controls	Bacteria 24 h; fungal endpoints 72 h or 3–7 d; release 6 h; cell viability 24/48 h	Nanoparticle-containing gel, Chi-MN-CHXNP-TE: reported MICs 0.016 µg/mL (*S. aureus*), 0.014 (*S. pseudintermedius*) and 1.22 (*M. pachydermatis*); *M. canis* not determined. Corresponding gel without nanoparticles: 0.20, 0.20, 1.02 and 0.04 µg/mL, respectively. Concentration basis for multicomponent MICs not explicitly defined. Cs gel alone showed no cytotoxicity under tested conditions; loaded-formulation effects cannot be assigned to Cs alone.	[82]
Drug, vaccine and biological delivery systems	Commercial Cs C84: DDA 84%, MW 421.8 kDa; C95: 95%, 455.2 kDa; M85/M91: DDA 85%/91%, MW NR	Four thermogelling Cs /β-glycerophosphate formulations, prepared from 1.8% (*w*/*w*) Cs solutions; final pH 7.15; single subcutaneous injection, 50 µL per hindpaw	Experimental animal study: adult male Wistar rats, approximately 250 g; *n* = 6–15/group across experiments	β-Glycerophosphate alone; parallel pharmacological-pretreatment groups	Acute measurements: 0–24 h; persistent inflammation reported at ≥28 d	At 5 h, C95/M91 produced less oedema than C84/M85 (*p* < 0.05), but all exceeded β-glycerophosphate alone. Dexamethasone suppressed acute oedema; inflammation persisted to ≥28 d, reported without displayed data.	[83]
Feed additives and dietary supplementation	Food-grade, GMP-certified chitosan; origin/supplier NR; DDA 90.34%; MW 250–500 kDa. Source abbreviation “COS” denotes chitosan, not oligosaccharides.	Chitosan, 100 mg/kg feed; corn-soybean basal diet. Day-6 oral challenge: 100 mL EHEC O157:H7, 1 × 10^9^ CFU/mL.	Randomised experimental feeding/challenge study: 108 weaned piglets; 3 groups, 18 pens/group, 2 pigs/pen; equal male/female ratio.	Unchallenged basal-diet control; EHEC-challenged basal-diet control	21 d	Days 6–14 FCR: 1.90 vs. 2.30 (challenged control), overall *p* = 0.001; pairwise *p* < 0.05. No significant final BW or feed-intake difference. Improved intestinal endpoints; bacterial shedding reduction not significant.	[89]
Feed additives and dietary supplementation	Commercial chitosan (HIMEDIA); biological origin, DDA and MW NR.	Chitosan, 360 mg/kg concentrate DM, administered orally before feeding; no additional carrier	Randomised experimental feeding study; female Jabal Akhdar goats. 18 animals, *n* = 9/group.	Basal diet without chitosan	42 d, after 14 d acclimation	Chitosan/control (mean ± SE): serum protein 69.04 ± 1.81/62.09 ± 2.10 g/L (*p* = 0.023); blood urea nitrogen 4.85 ± 0.22/6.14 ± 0.19 mmol/L (*p* < 0.001). Reduced ruminal ammonia; fermentation shifted towards propionate.	[90]
Feed additives and dietary supplementation	Food-grade, GMP-certified chitosan; origin/supplier NR; DDA 90.34%; MW 250–500 kDa. Source abbreviation “COS” denotes chitosan, not oligosaccharides.	Chitosan, 100 mg/kg feed; corn-soybean basal diet. Day-6 oral challenge: 100 mL EHEC O157:H7, 1 × 10^9^ CFU/mL.	Randomised experimental feeding/challenge study: 108 weaned piglets; 3 groups, 18 pens/group, 2 pigs/pen; equal male/female ratio.	Unchallenged basal-diet control; EHEC-challenged basal-diet control	21 d	Days 6–14 FCR: 1.90 vs. 2.30 (challenged control), overall *p* = 0.001; pairwise *p* < 0.05. No significant final BW or feed-intake difference. Improved intestinal endpoints; bacterial shedding reduction not significant.	[91]

Cs, chitosan; COS, chitosan oligosaccharides; DDA, degree of deacetylation; MW, molecular weight; CF, chitosan fibre dressing; CHX, chlorhexidine; MN, miconazole; EDTA, ethylenediaminetetraacetic acid; TE, Tris-EDTA; TPP, tripolyphosphate; AgNPs, silver nanoparticles; GPTMS, 3-glycidoxypropyltrimethoxysilane; DSP, dibasic sodium phosphate; DCS, catechol-modified chitosan; PEGSH, aldehyde-functionalised PEG/sebacic-acid polymer; PRP, platelet-rich plasma; α-SMA, alpha-smooth muscle actin; EGF, epidermal growth factor; HA, hyaluronic acid; P, pectin; MTA, mineral trioxide aggregate; MIC, minimum inhibitory concentration; BCI, blood clotting index; HUVEC, human umbilical vein endothelial cells; MRSA, methicillin-resistant *Staphylococcus aureus*; PBS, phosphate-buffered saline; IV, intravenous; IP, intraperitoneal; GMP, Good Manufacturing Practice; EHEC, enterohaemorrhagic *Escherichia coli*; CFU, colony-forming units; DM, dry matter; SD, standard deviation; IQR, interquartile range; NR, not reported; n, sample size; *w*/*w*, weight/weight; *w*/*v*, weight/volume; d, days; h, hours; min, minutes; s, seconds.

## Data Availability

The raw data supporting the conclusions of this article will be made available by the authors on request.

## Abbreviations

The following abbreviations are used in this manuscript:

BSF	black soldier fly
*H. illucens*	*Hermetia illucens*
*T. molitor*	*Tenebrio molitor*
ADF	acid detergent fibre
ADL	acid detergent lignin
NMR	nuclear magnetic resonance
^13^C NMR	carbon-13 nuclear magnetic resonance spectroscopy
M_n_	number-average molecular weight
M*w*	weight-average molecular weight
SEC	size-exclusion chromatography
RI	refractive index
SEC-MALS-RI	size-exclusion chromatography coupled with multi-angle light scattering and refractive index detection
MALDI-TOF MS	matrix-assisted laser desorption/ionisation time-of-flight mass spectrometry
HMWC	high-molecular-weight chitosan
MMWC	medium-molecular-weight chitosan
LMWC	low-molecular-weight chitosan
DA	degree of acetylation
ATR-FTIR	attenuated total reflectance Fourier-transform infrared spectroscopy
XRD	X-ray diffraction
TEM	transmission electron microscopy
DSC	differential scanning calorimetry
ATCC	American Type Culture Collection
DMEM	Dulbecco’s modified Eagle medium
TLR	Toll-like receptor
NF-κB	nuclear factor kappa B
Th1/Th2	T helper type 1/type 2
SE	standard error
EFSA	European Food Safety Authority
ICP-MS	inductively coupled plasma mass spectrometry
PVA	poly(vinyl alcohol)
EU	European Union
EC	European Community
